# Supplementary figures and images for: Translation of dipeptide repeat proteins in C9ORF72 ALS/FTD through unique and redundant AUG initiation codons
Source: eLife. 2023 Sep 7;12:e83189. doi: 10.7554/eLife.83189 (PMC10541178; doi:10.7554/eLife.83189)

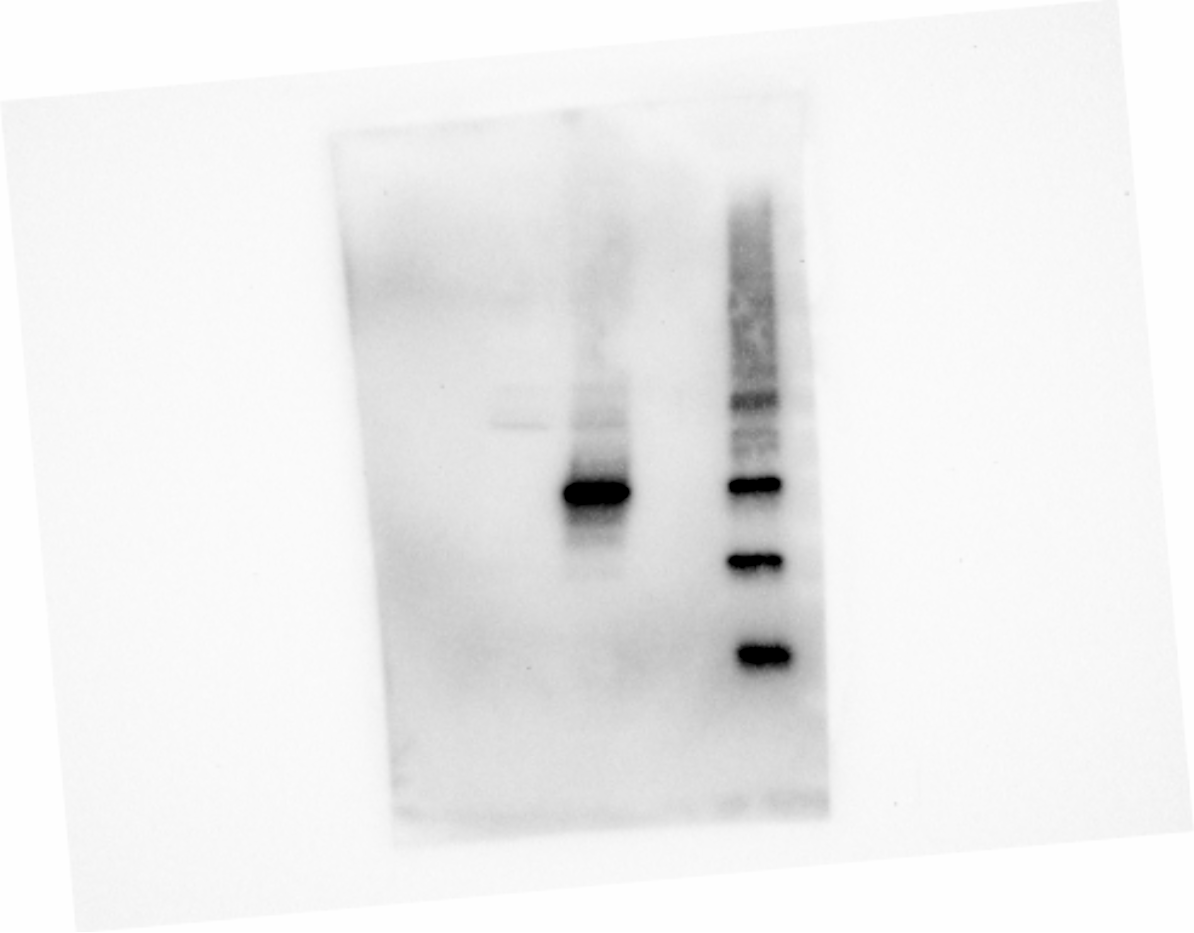

Supplement: Figure 1—source data 1. — Figures with the uncropped blots are clearly labeled with the relevant bands. [file elife-83189-fig1-data1.zip › Figure 1-source data 1/Fig. 1D NSC34 Poly-PR.tif]

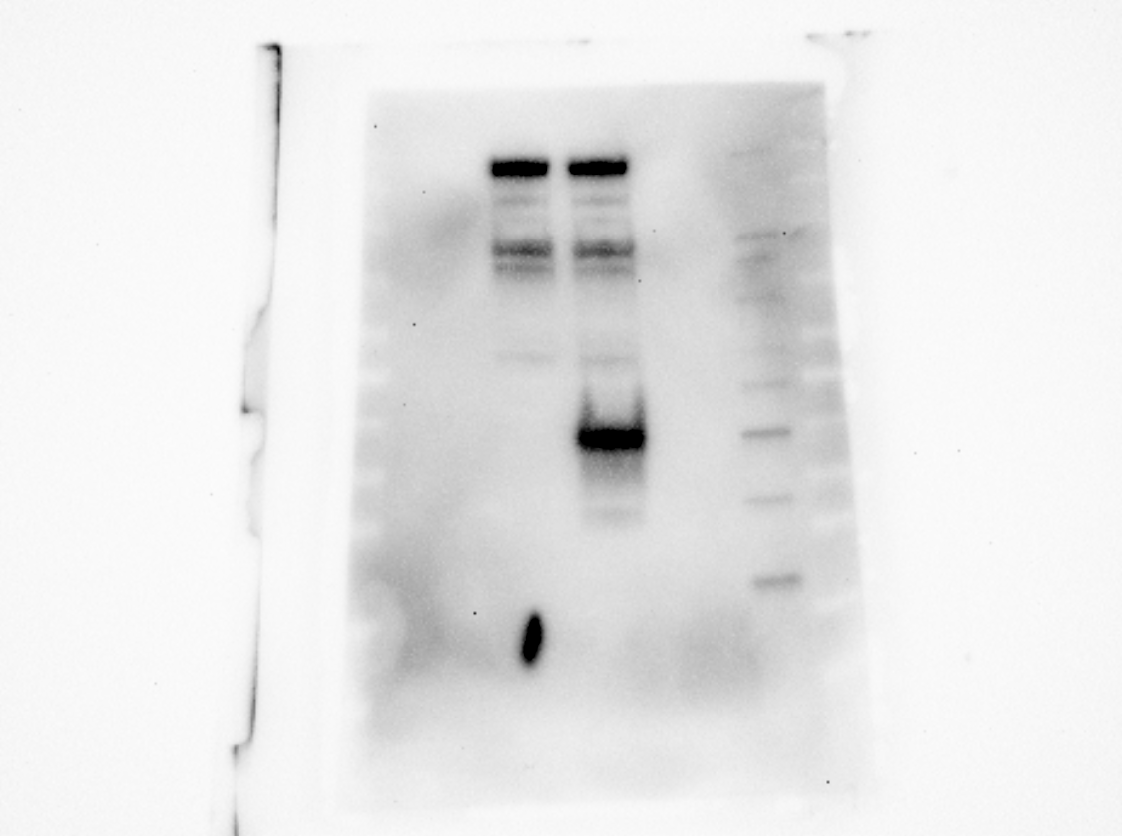

Supplement: Figure 1—source data 1. — Figures with the uncropped blots are clearly labeled with the relevant bands. [file elife-83189-fig1-data1.zip › Figure 1-source data 1/Fig. 1E NSC34 Poly-PG.tiff]

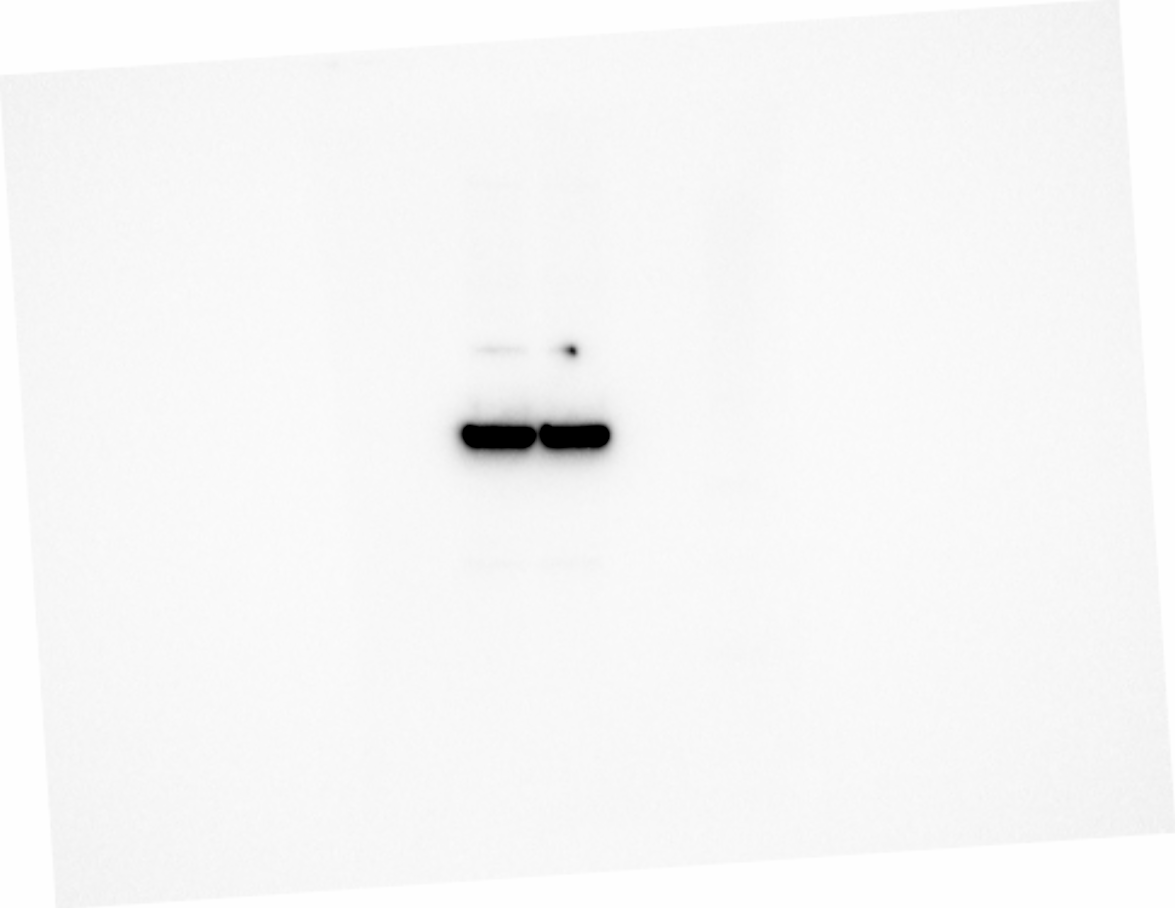

Supplement: Figure 1—source data 1. — Figures with the uncropped blots are clearly labeled with the relevant bands. [file elife-83189-fig1-data1.zip › Figure 1-source data 1/Fig. 1D NSC34 a-tubulin.tif]

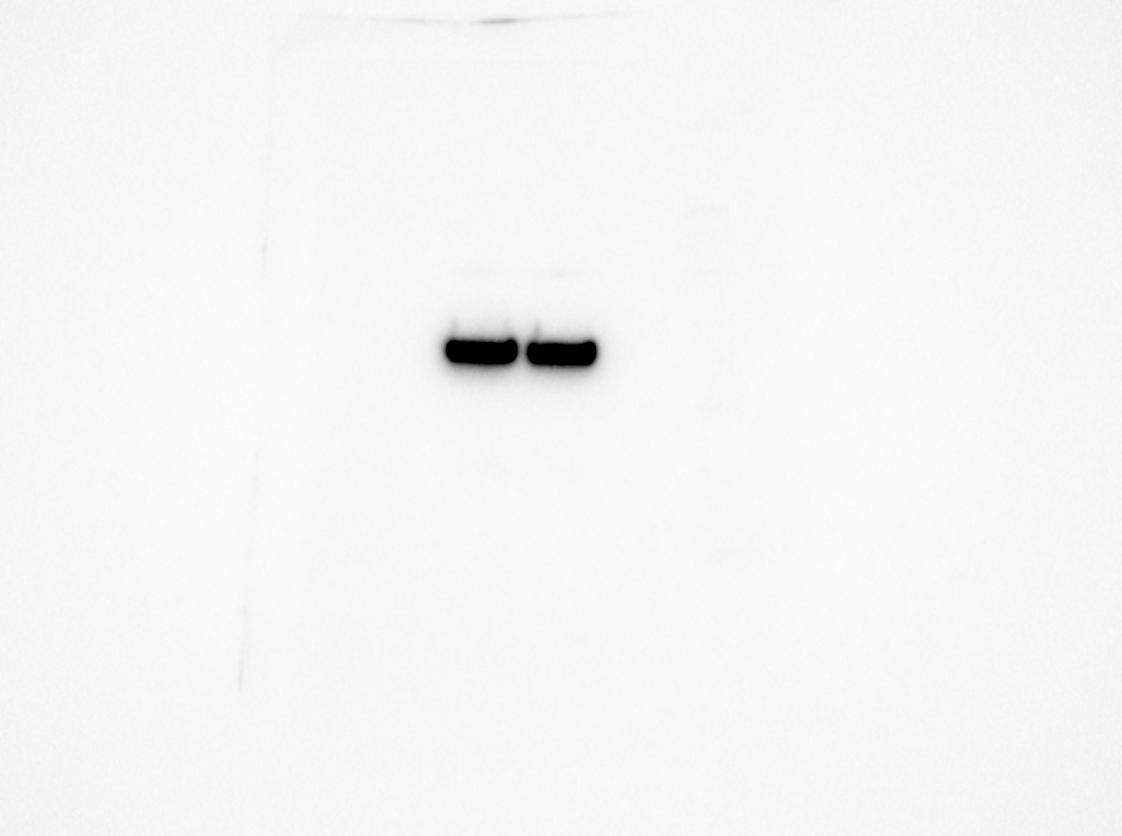

Supplement: Figure 1—source data 1. — Figures with the uncropped blots are clearly labeled with the relevant bands. [file elife-83189-fig1-data1.zip › Figure 1-source data 1/Fig. 1E NSC34 a-tubulin.tif]

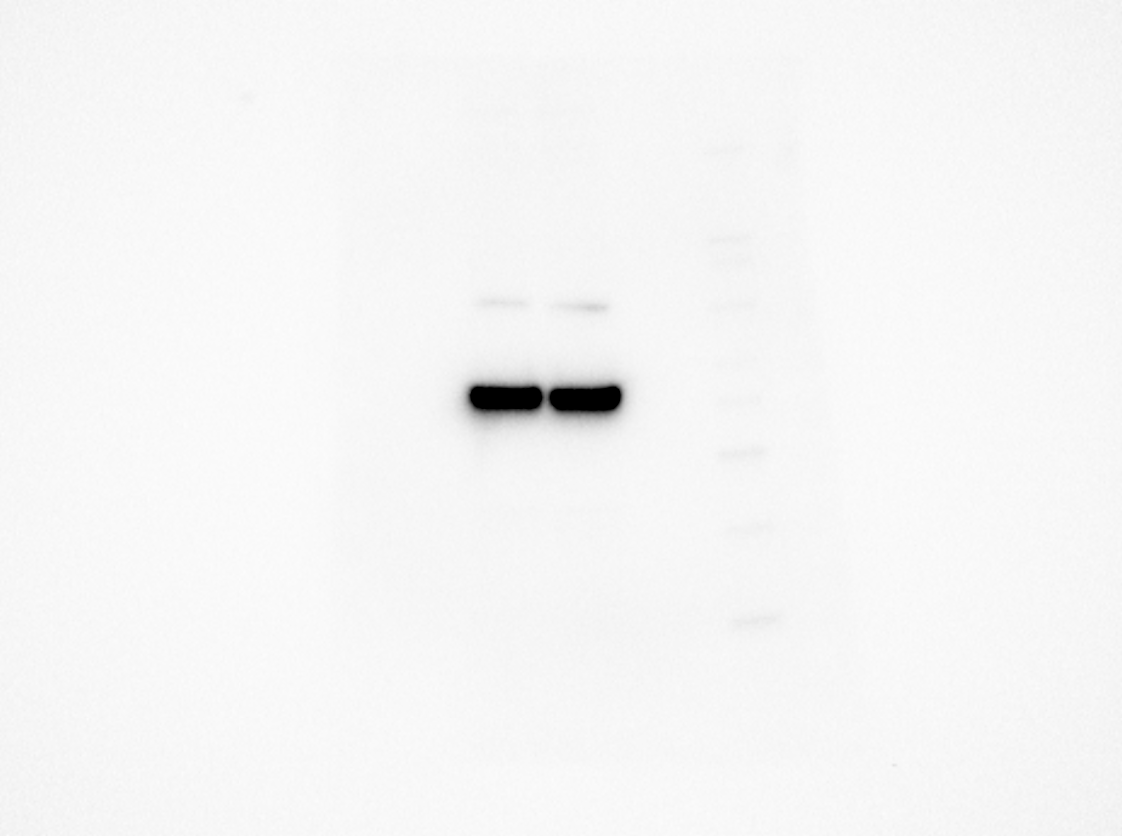

Supplement: Figure 1—source data 1. — Figures with the uncropped blots are clearly labeled with the relevant bands. [file elife-83189-fig1-data1.zip › Figure 1-source data 1/Fig. 1E HEK293 a-tubulin.tif]

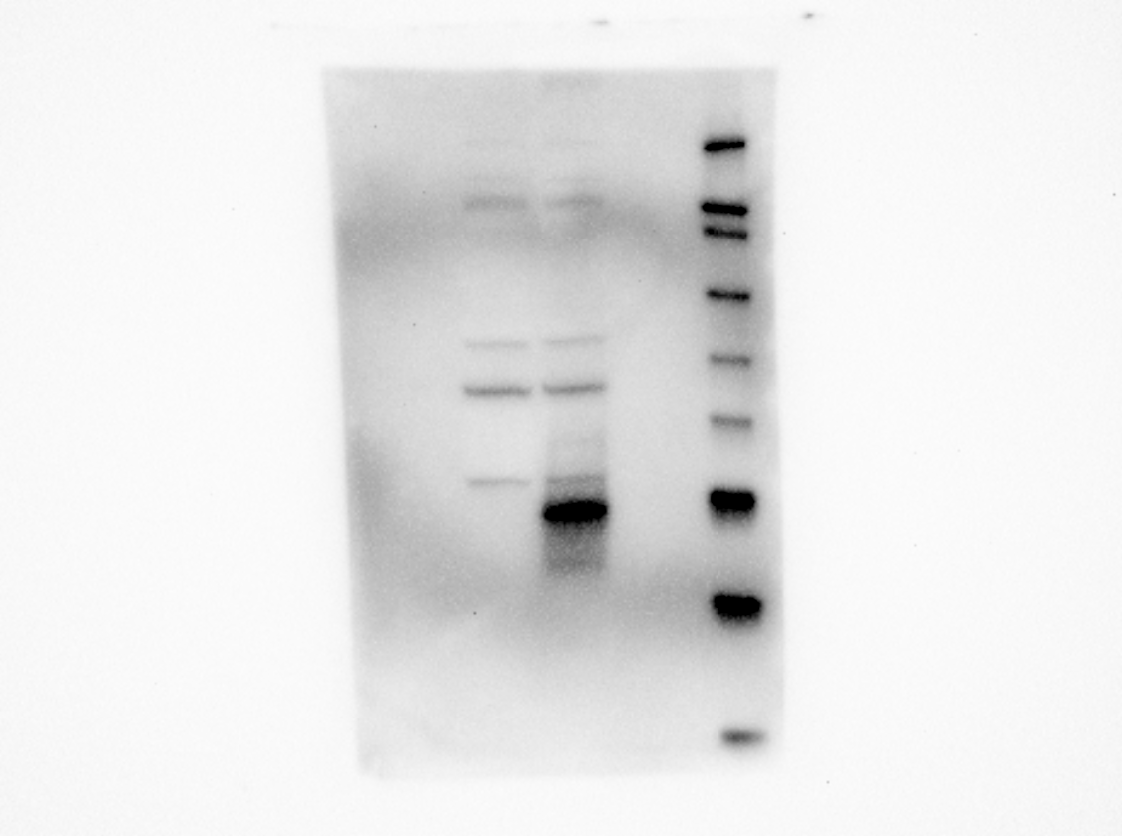

Supplement: Figure 1—source data 1. — Figures with the uncropped blots are clearly labeled with the relevant bands. [file elife-83189-fig1-data1.zip › Figure 1-source data 1/Fig. 1D HEK293 Poly-PR.tiff]

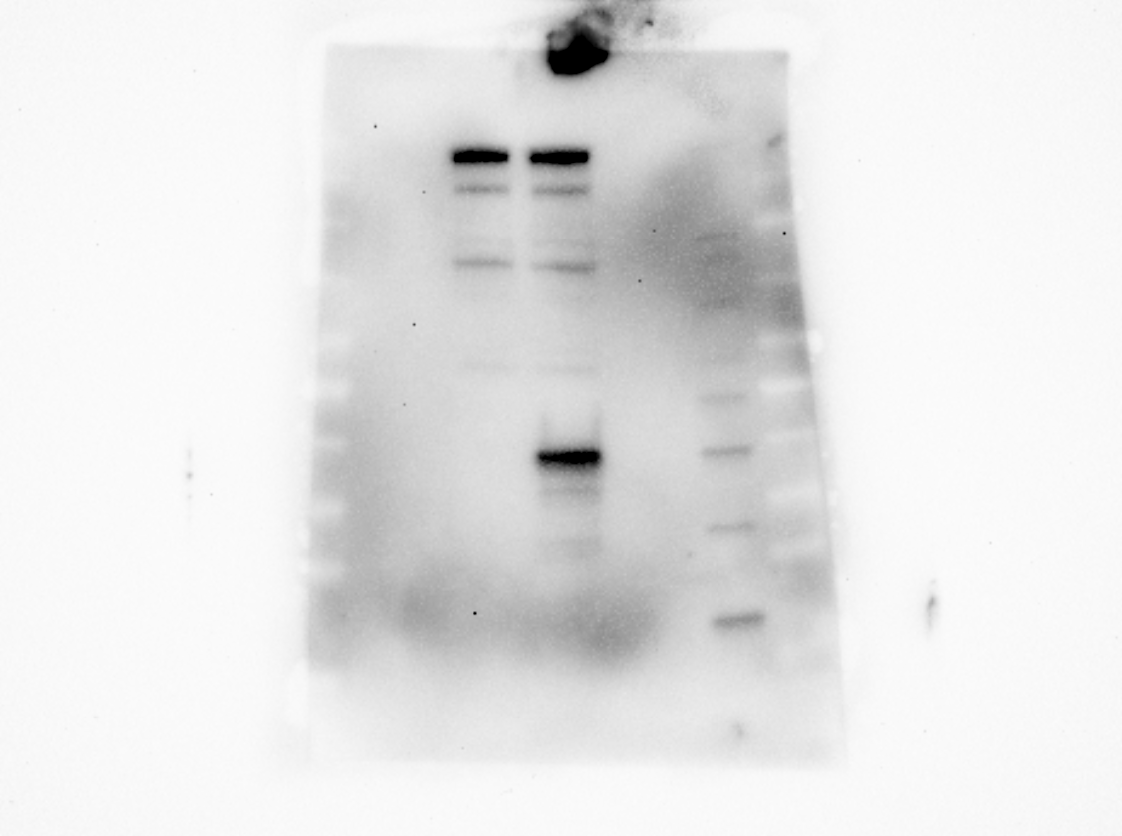

Supplement: Figure 1—source data 1. — Figures with the uncropped blots are clearly labeled with the relevant bands. [file elife-83189-fig1-data1.zip › Figure 1-source data 1/Fig. 1E HEK293 Poly-PG.tiff]

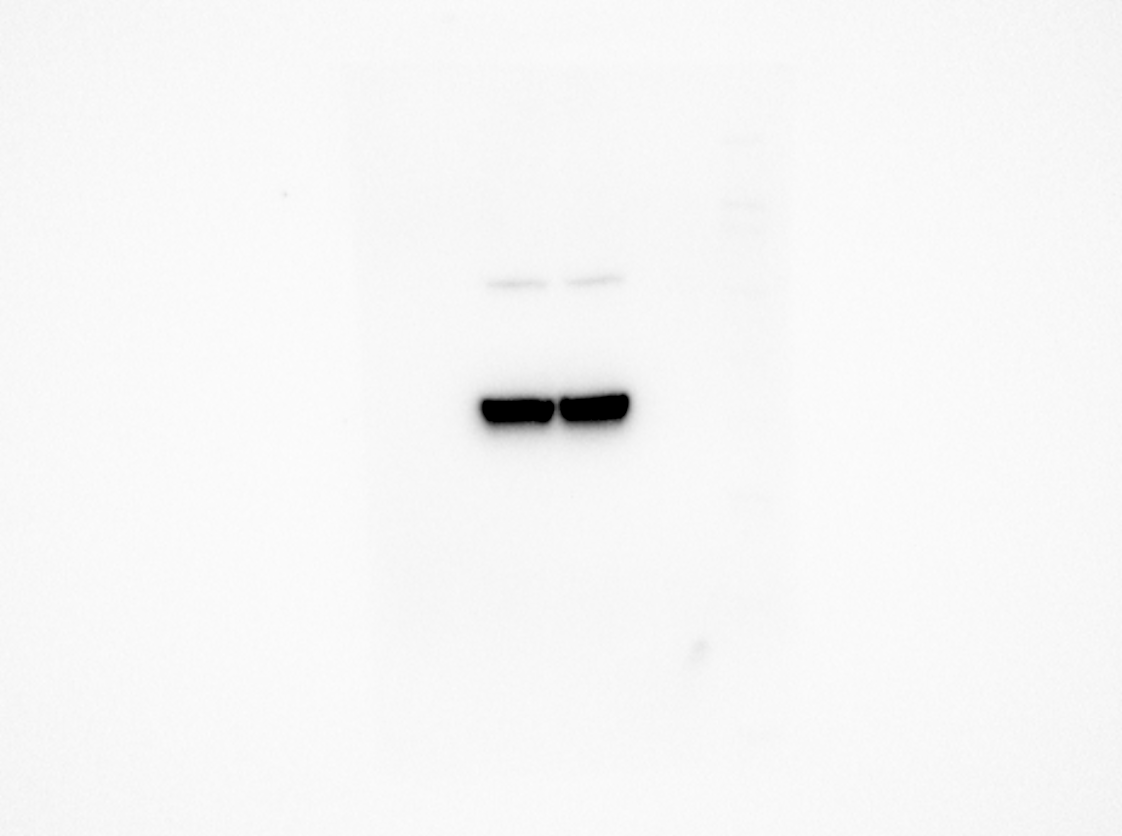

Supplement: Figure 1—source data 1. — Figures with the uncropped blots are clearly labeled with the relevant bands. [file elife-83189-fig1-data1.zip › Figure 1-source data 1/Fig. 1D HEK293 a-tubulin.tif]

Figure 1D, HEK293

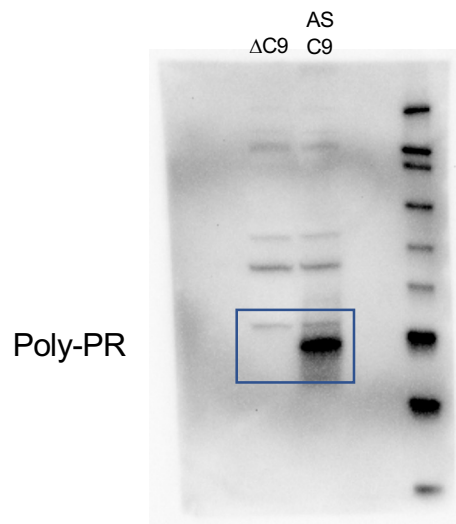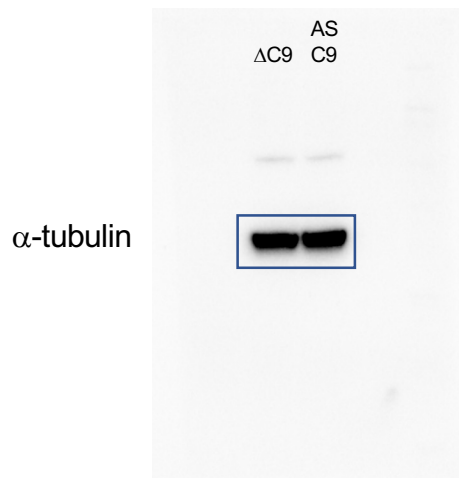

Figure 1E, HEK293

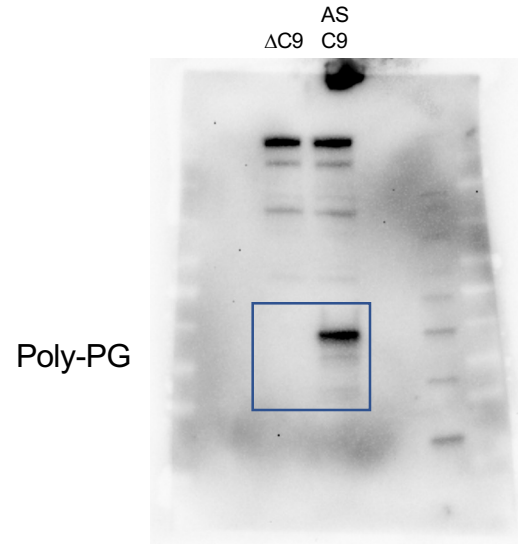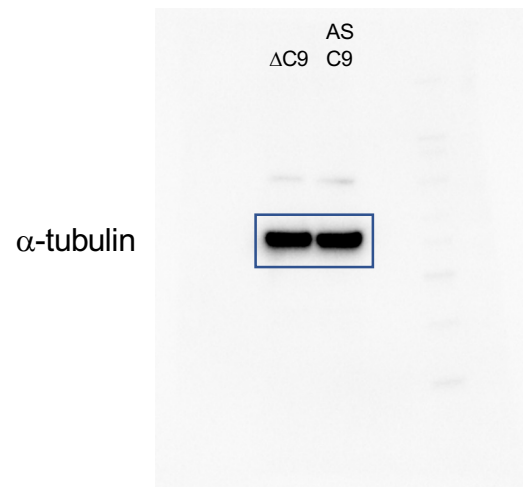

Figure 1D, NSC34

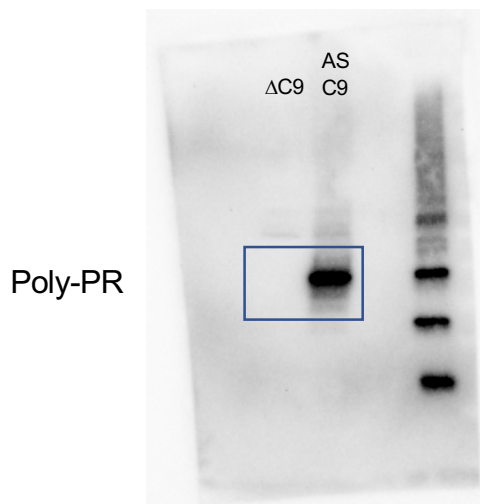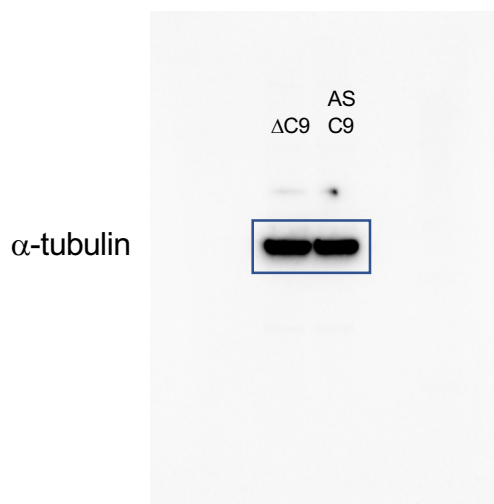

Figure 1E, NSC34

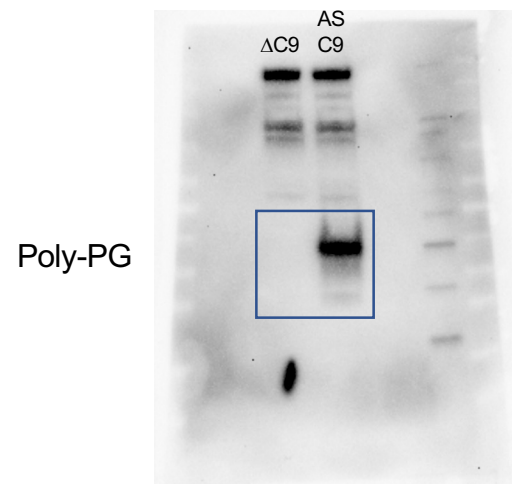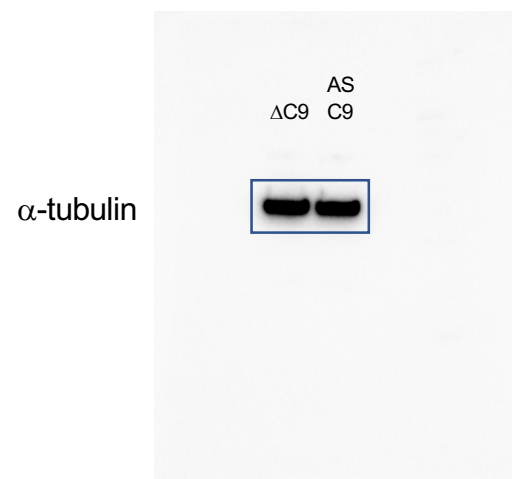

Supplement: Figure 1—source data 1. — Figures with the uncropped blots are clearly labeled with the relevant bands. [file elife-83189-fig1-data1.zip › Figure 1-source data 1/Figure 1-source data.pdf]

Figure 1 – figure supplement 1B

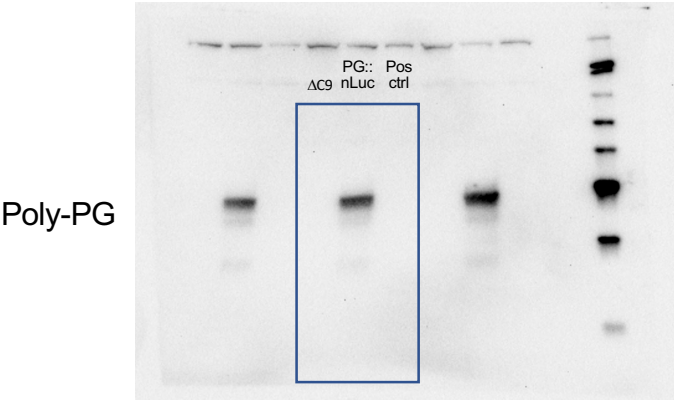

Figure 1 – figure supplement 1C

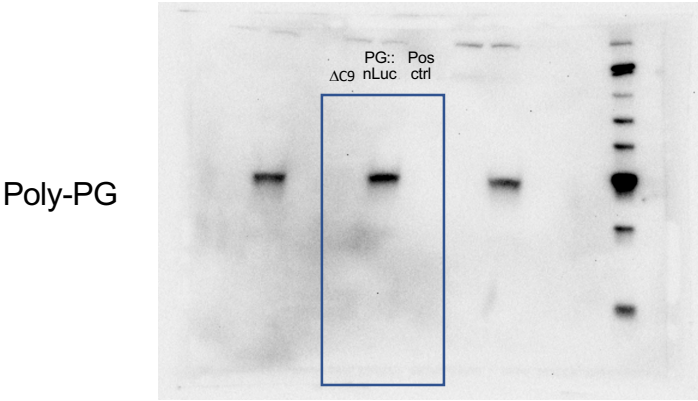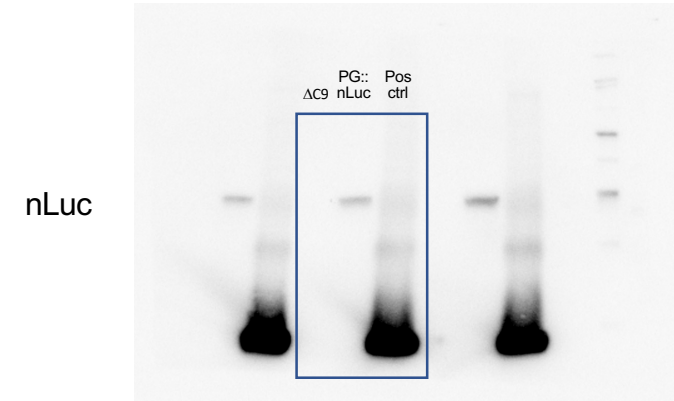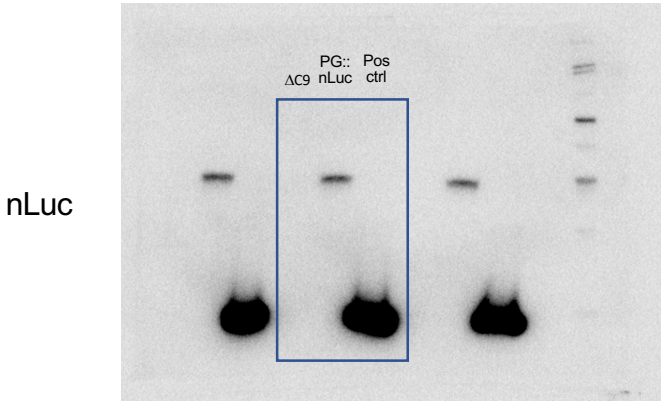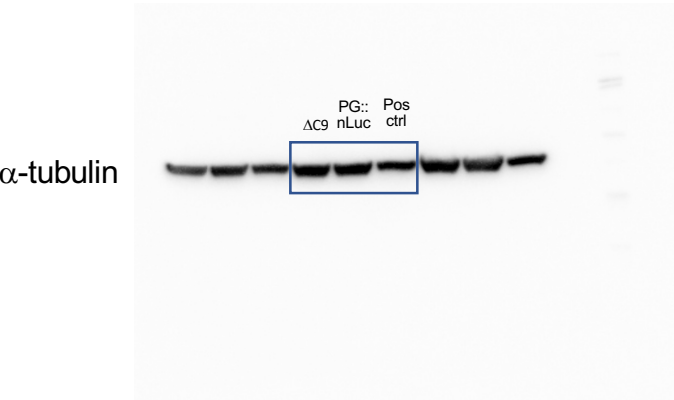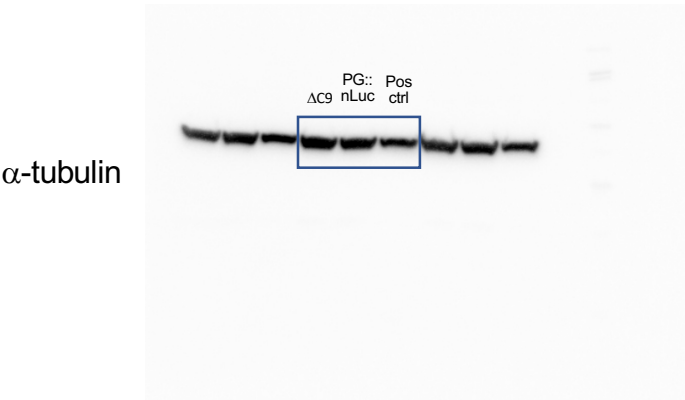

Supplement: Figure 1—figure supplement 1—source data 1. — Figures with the uncropped blots are clearly labeled with the relevant bands. [file elife-83189-fig1-figsupp1-data1.zip › Figure 1-figure supplement 1-source data 1/Figure 1 - figure supplement 1-source data labelled.pdf]

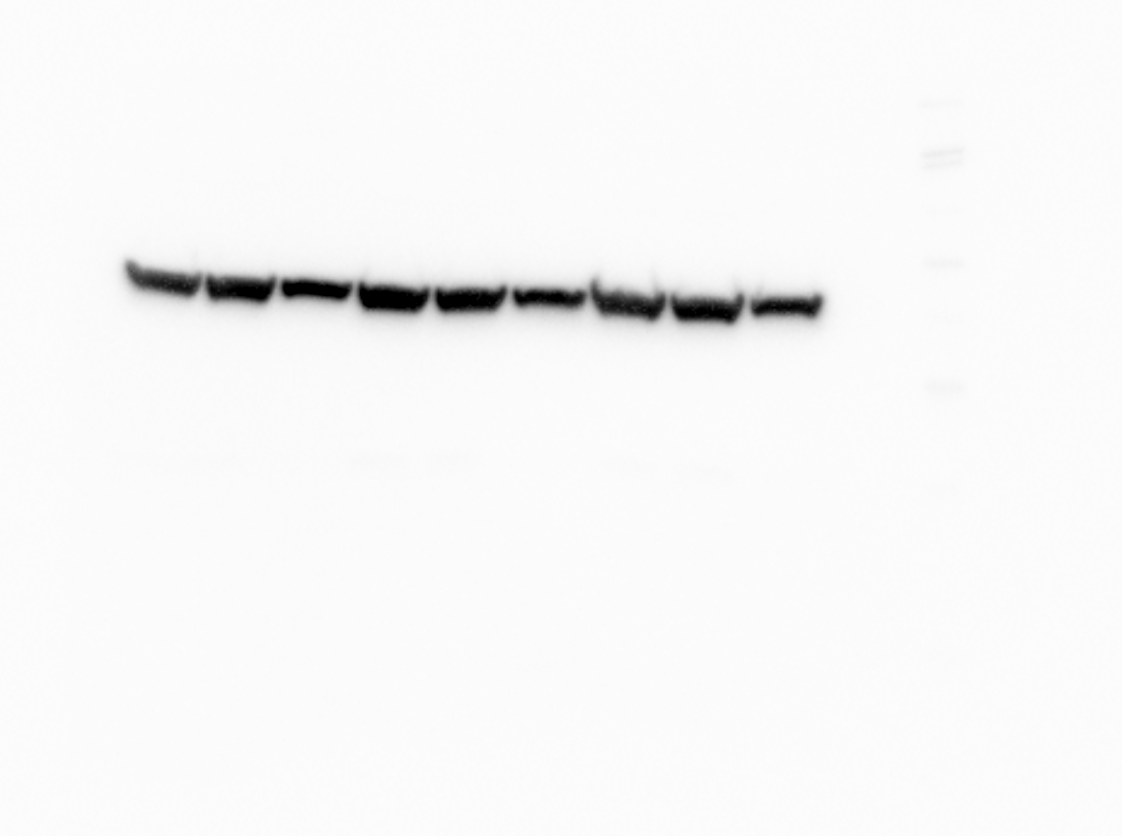

Supplement: Figure 1—figure supplement 1—source data 1. — Figures with the uncropped blots are clearly labeled with the relevant bands. [file elife-83189-fig1-figsupp1-data1.zip › Figure 1-figure supplement 1-source data 1/Fig. 1 Suppl 1C a-tubulin.tiff]

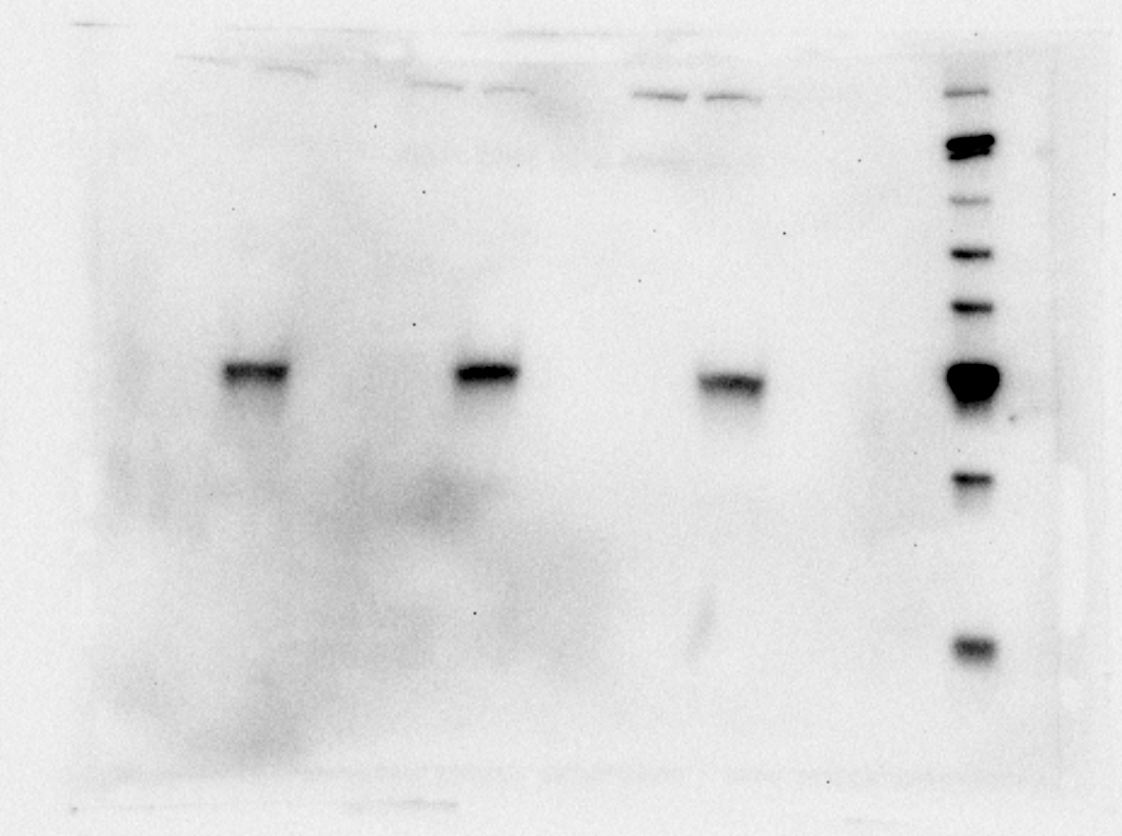

Supplement: Figure 1—figure supplement 1—source data 1. — Figures with the uncropped blots are clearly labeled with the relevant bands. [file elife-83189-fig1-figsupp1-data1.zip › Figure 1-figure supplement 1-source data 1/Fig. 1 Suppl 1C Poly-PG.tiff]

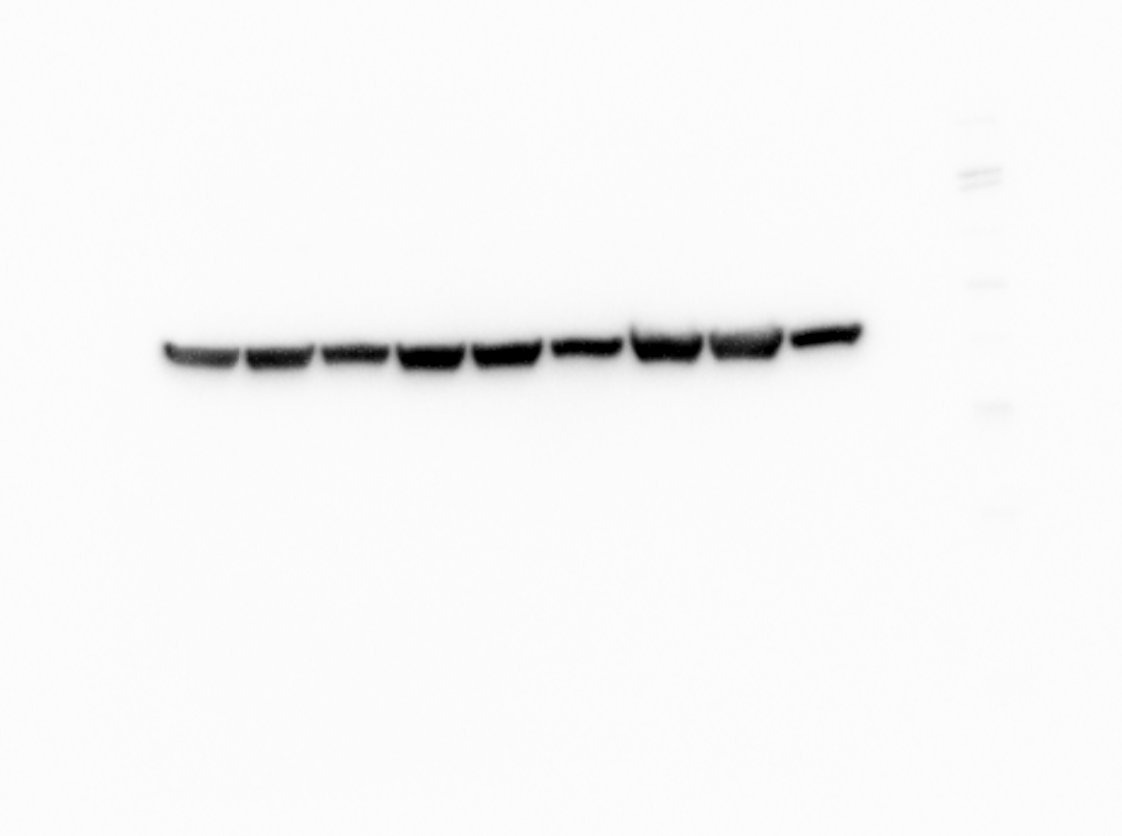

Supplement: Figure 1—figure supplement 1—source data 1. — Figures with the uncropped blots are clearly labeled with the relevant bands. [file elife-83189-fig1-figsupp1-data1.zip › Figure 1-figure supplement 1-source data 1/Fig. 1 Suppl 1B a-tubulin.tiff]

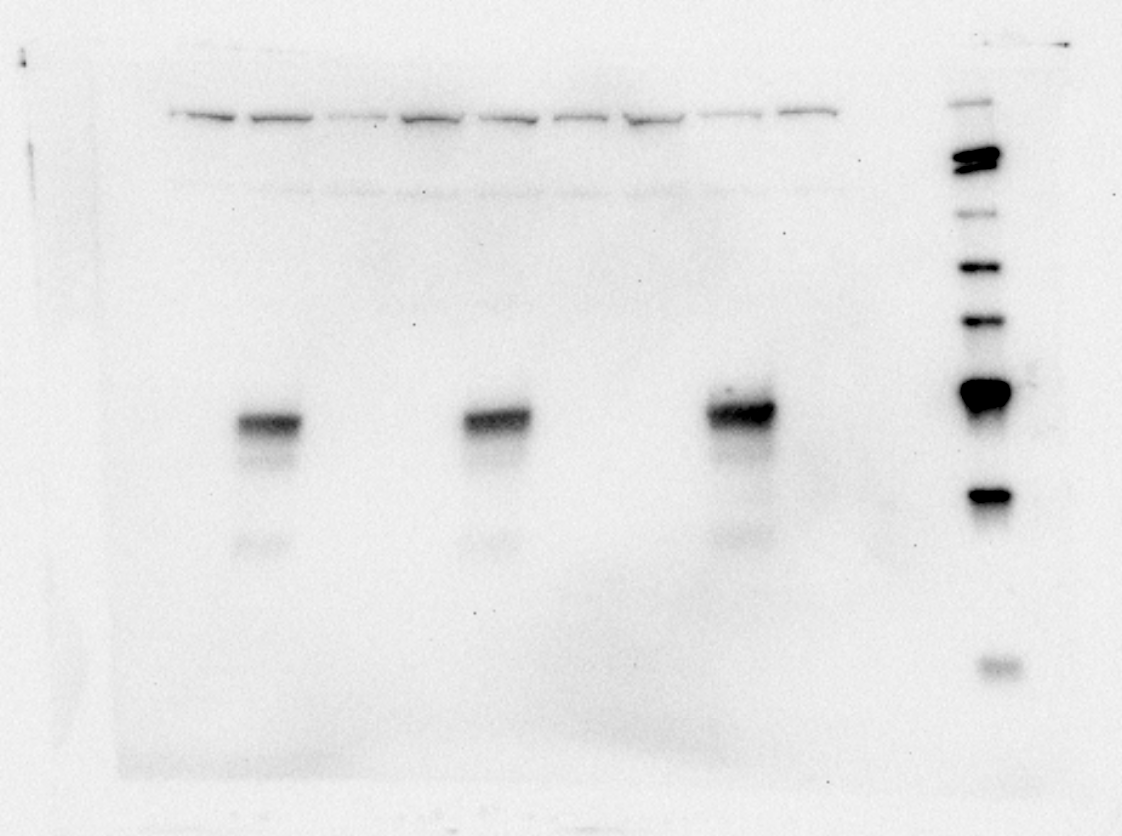

Supplement: Figure 1—figure supplement 1—source data 1. — Figures with the uncropped blots are clearly labeled with the relevant bands. [file elife-83189-fig1-figsupp1-data1.zip › Figure 1-figure supplement 1-source data 1/Fig. 1 Suppl 1B Poly-PG.tiff]

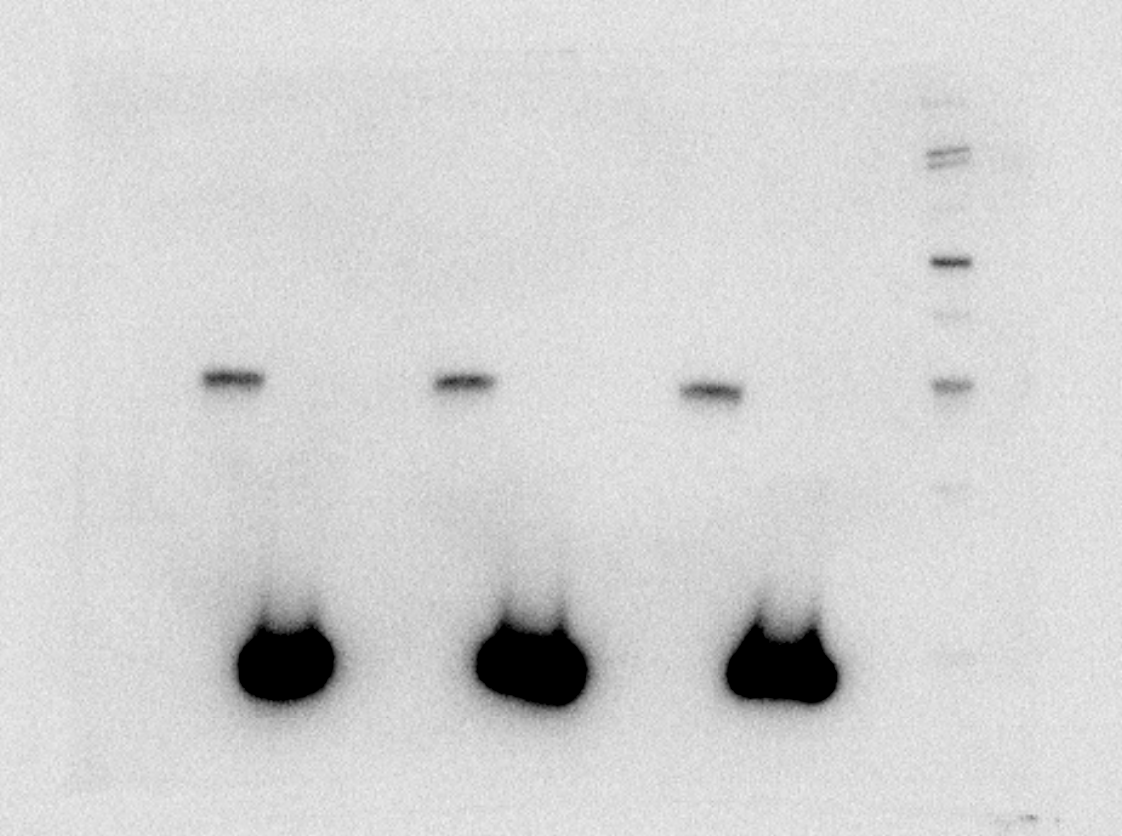

Supplement: Figure 1—figure supplement 1—source data 1. — Figures with the uncropped blots are clearly labeled with the relevant bands. [file elife-83189-fig1-figsupp1-data1.zip › Figure 1-figure supplement 1-source data 1/Fig. 1 Suppl 1C nLuc.tiff]

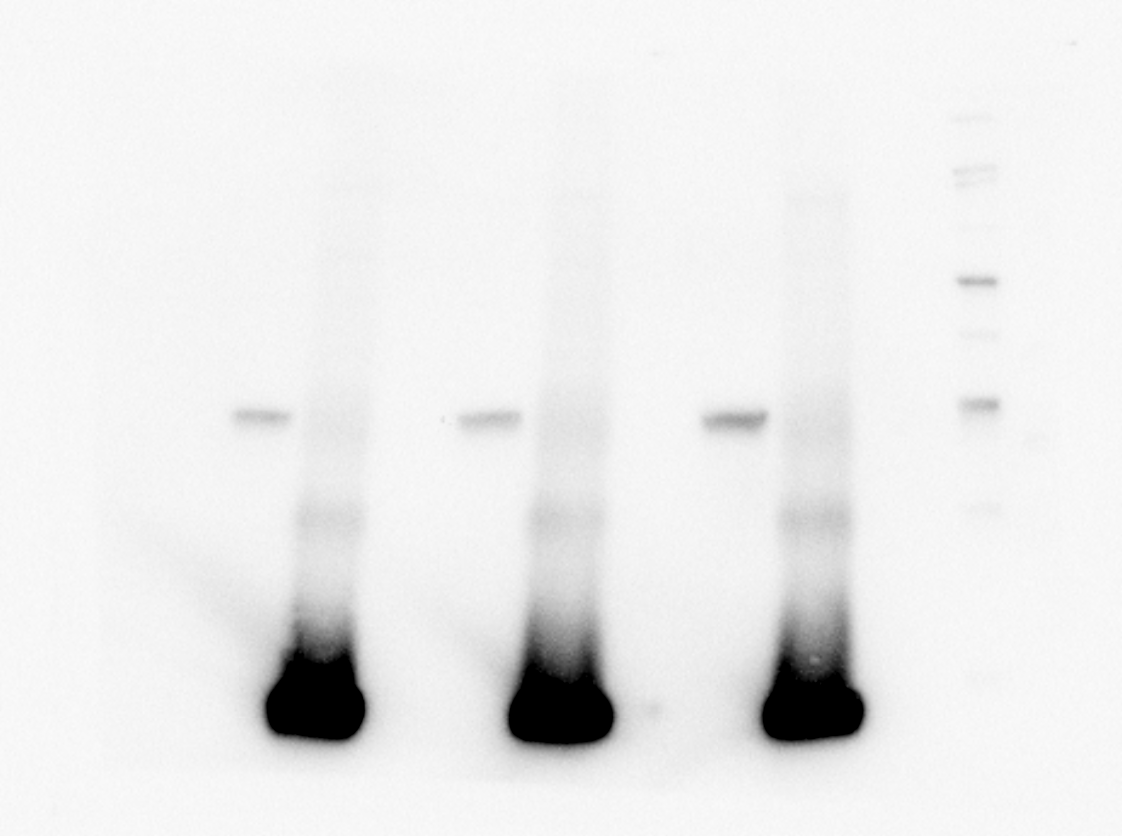

Supplement: Figure 1—figure supplement 1—source data 1. — Figures with the uncropped blots are clearly labeled with the relevant bands. [file elife-83189-fig1-figsupp1-data1.zip › Figure 1-figure supplement 1-source data 1/Fig. 1 Suppl 1B nLuc.tiff]

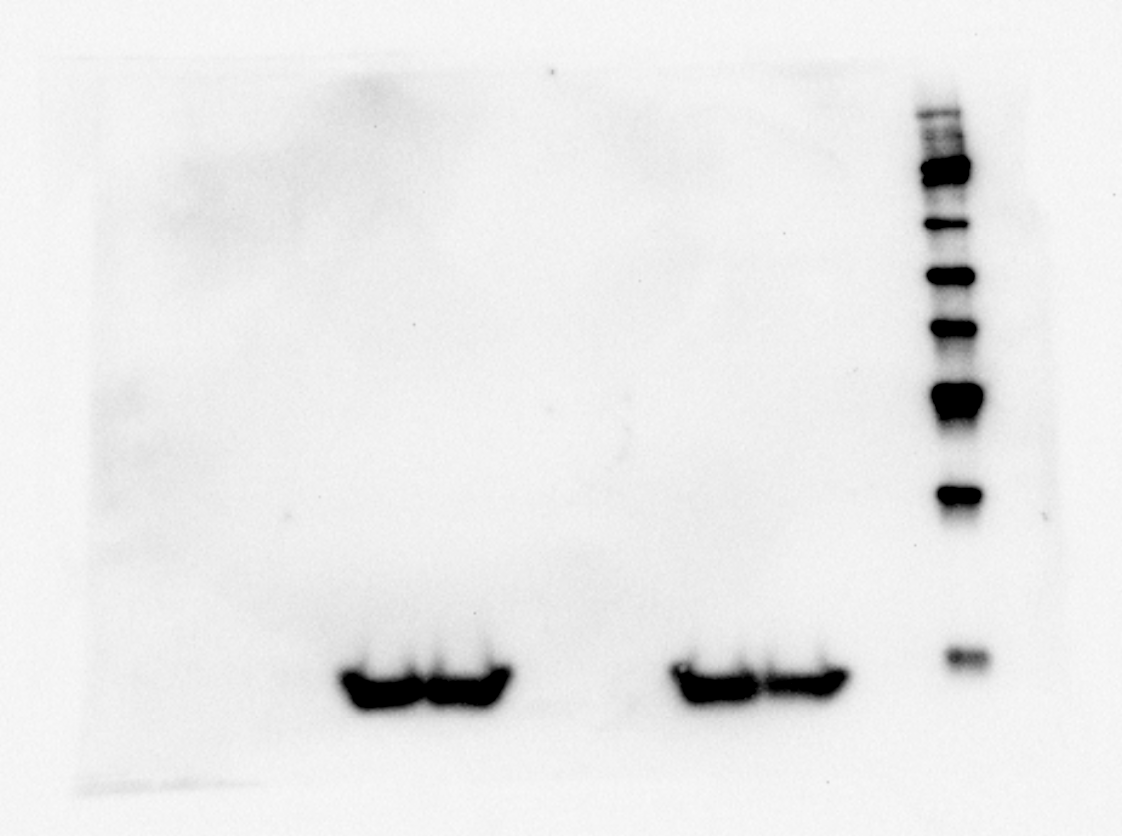

Supplement: Figure 1—figure supplement 2—source data 1. — Figures with the uncropped blots are clearly labeled with the relevant bands. [file elife-83189-fig1-figsupp2-data1.zip › Figure 1-figure supplement 2-source data 1/Fig.1 Suppl 2G H3K4me2.tiff]

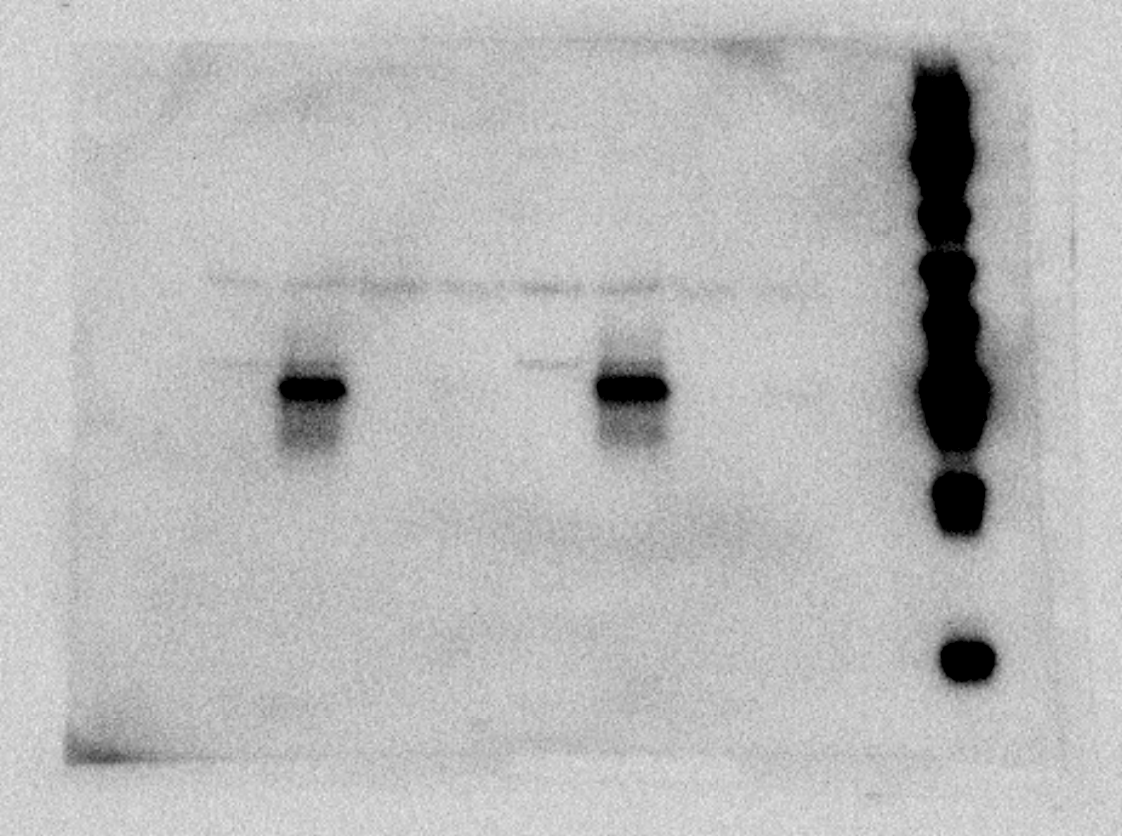

Supplement: Figure 1—figure supplement 2—source data 1. — Figures with the uncropped blots are clearly labeled with the relevant bands. [file elife-83189-fig1-figsupp2-data1.zip › Figure 1-figure supplement 2-source data 1/Fig. 1 Suppl 2B Poly-PR.tiff]

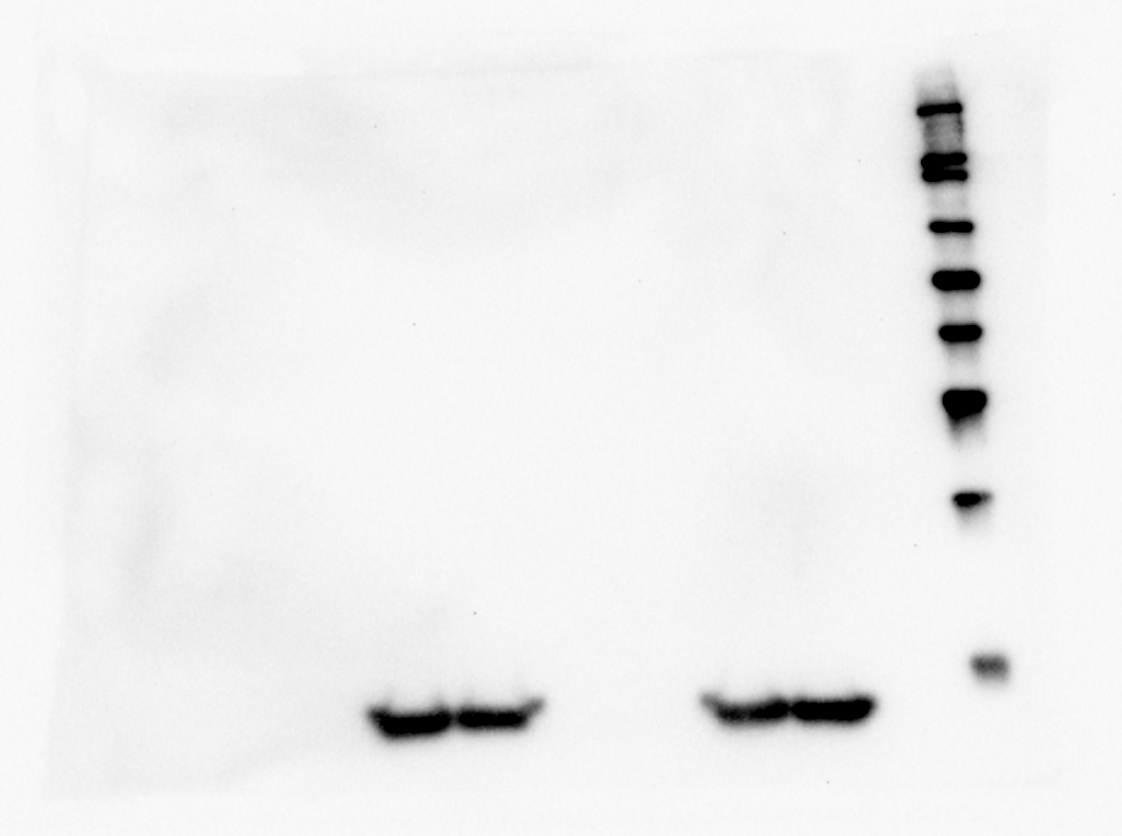

Supplement: Figure 1—figure supplement 2—source data 1. — Figures with the uncropped blots are clearly labeled with the relevant bands. [file elife-83189-fig1-figsupp2-data1.zip › Figure 1-figure supplement 2-source data 1/Fig. 1 Suppl 2F H3K4me2.tiff]

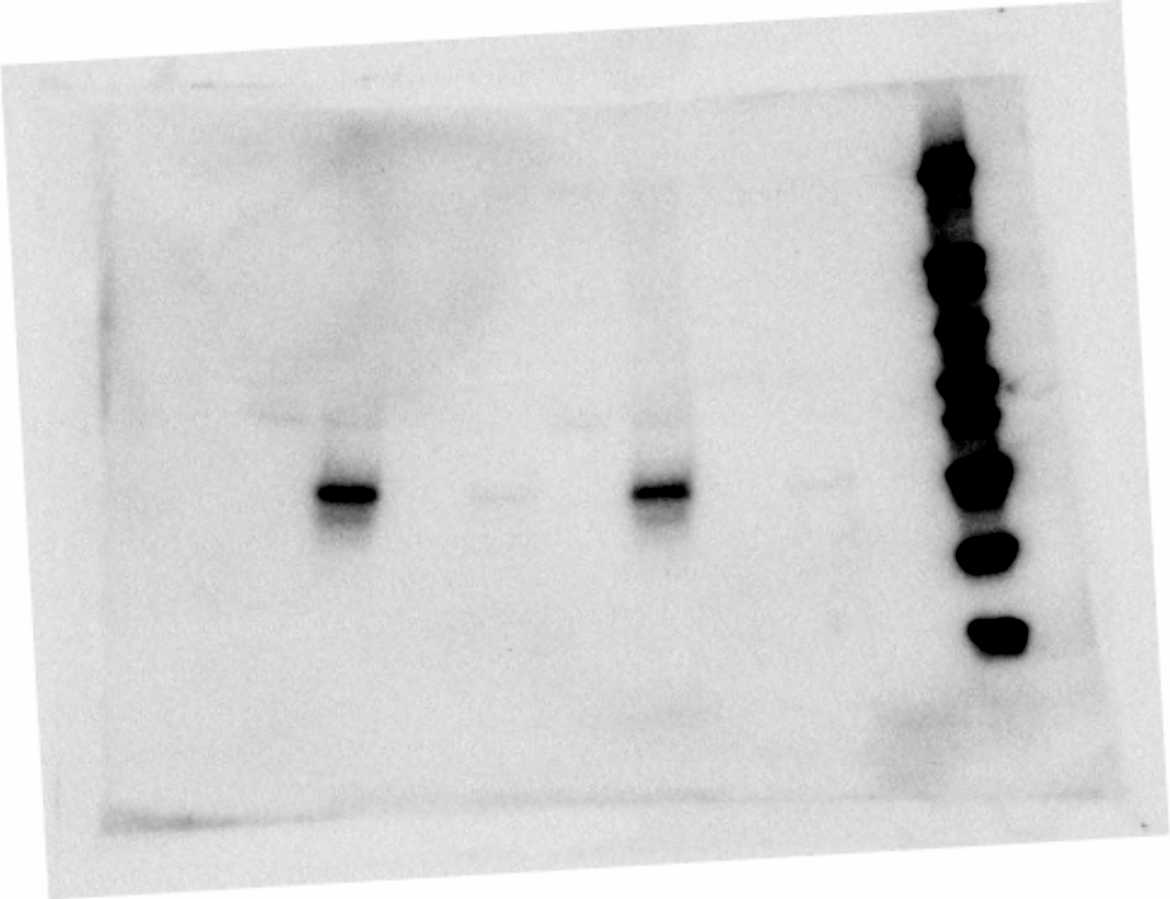

Supplement: Figure 1—figure supplement 2—source data 1. — Figures with the uncropped blots are clearly labeled with the relevant bands. [file elife-83189-fig1-figsupp2-data1.zip › Figure 1-figure supplement 2-source data 1/Fig. 1 Suppl 2C Poly-PR.tiff]

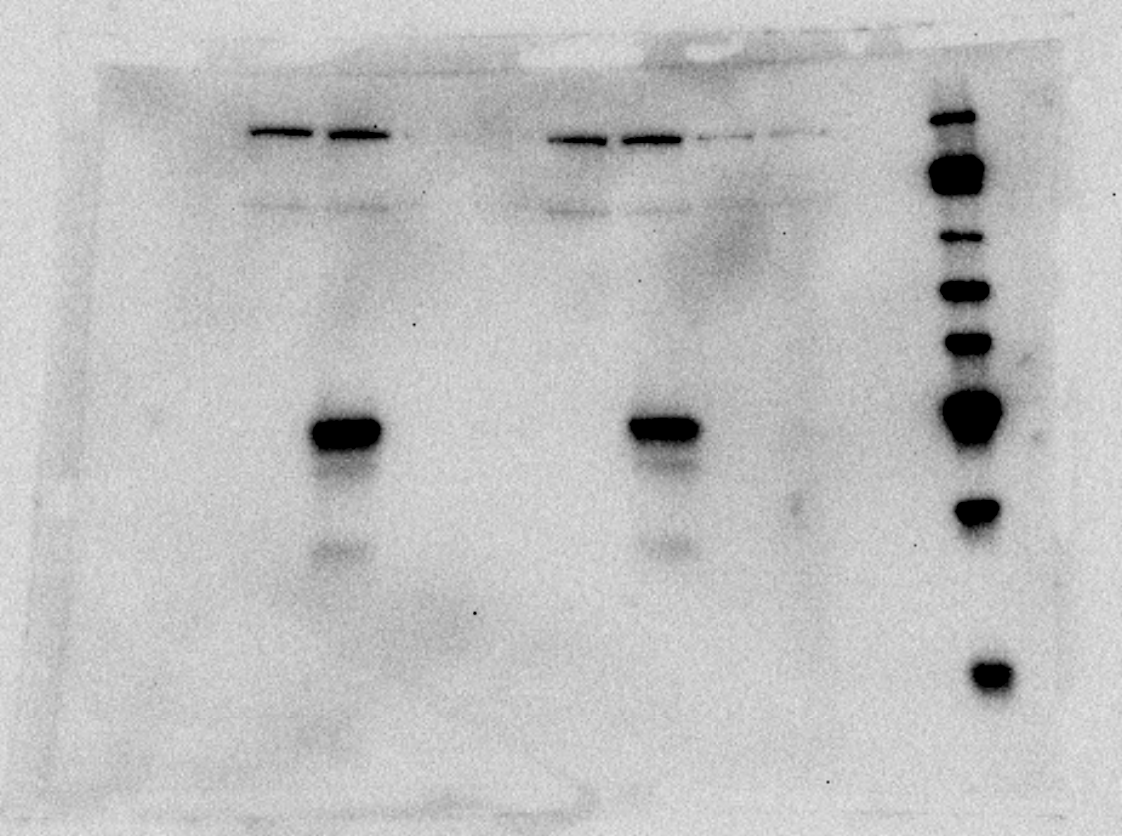

Supplement: Figure 1—figure supplement 2—source data 1. — Figures with the uncropped blots are clearly labeled with the relevant bands. [file elife-83189-fig1-figsupp2-data1.zip › Figure 1-figure supplement 2-source data 1/Fig. 1 Suppl 2F Poly-PG.tiff]

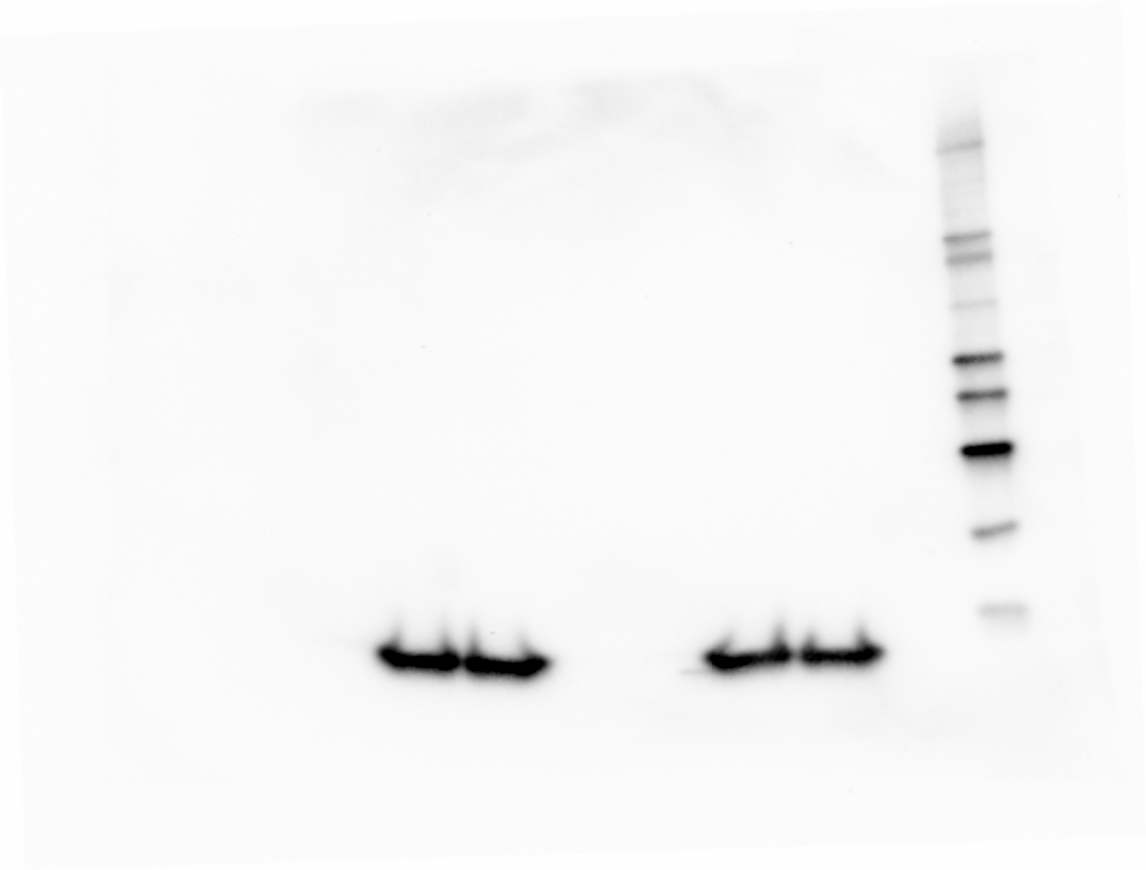

Supplement: Figure 1—figure supplement 2—source data 1. — Figures with the uncropped blots are clearly labeled with the relevant bands. [file elife-83189-fig1-figsupp2-data1.zip › Figure 1-figure supplement 2-source data 1/Fig. 1 Suppl 2C H3K4me2.tiff]

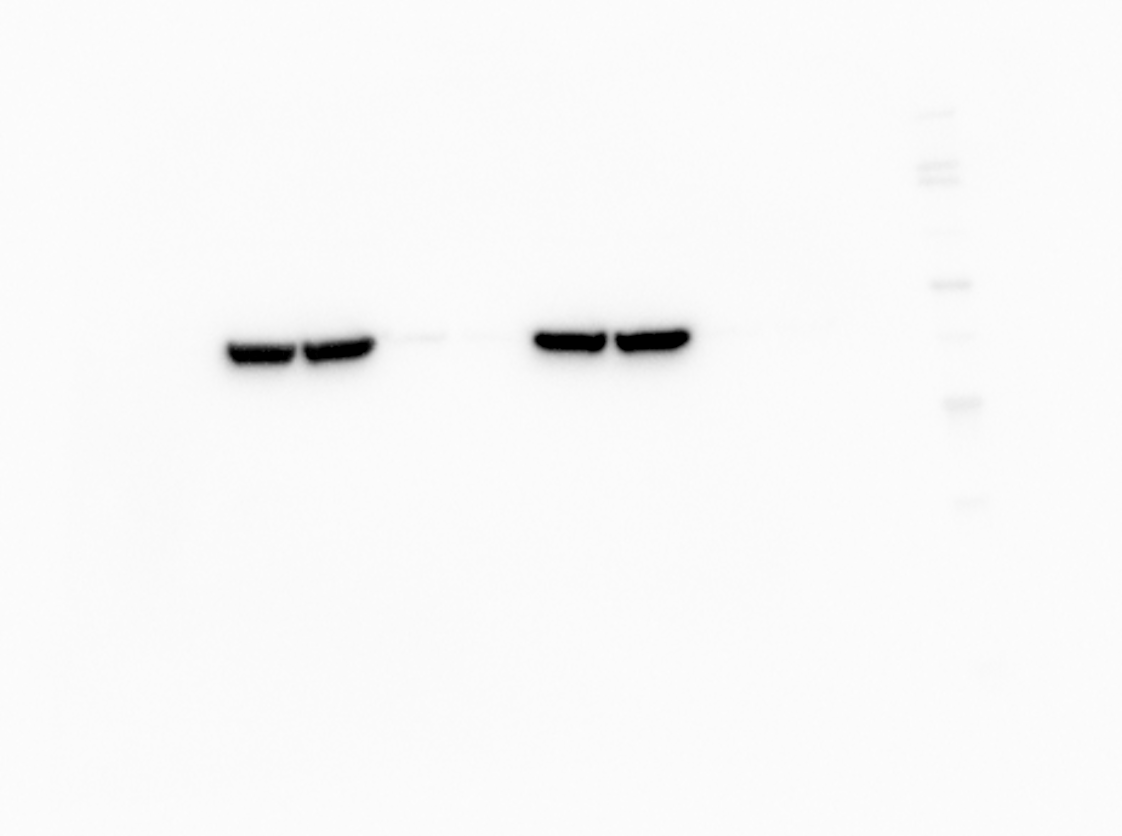

Supplement: Figure 1—figure supplement 2—source data 1. — Figures with the uncropped blots are clearly labeled with the relevant bands. [file elife-83189-fig1-figsupp2-data1.zip › Figure 1-figure supplement 2-source data 1/Fig. 1 Suppl 2F a-tubulin.tiff]

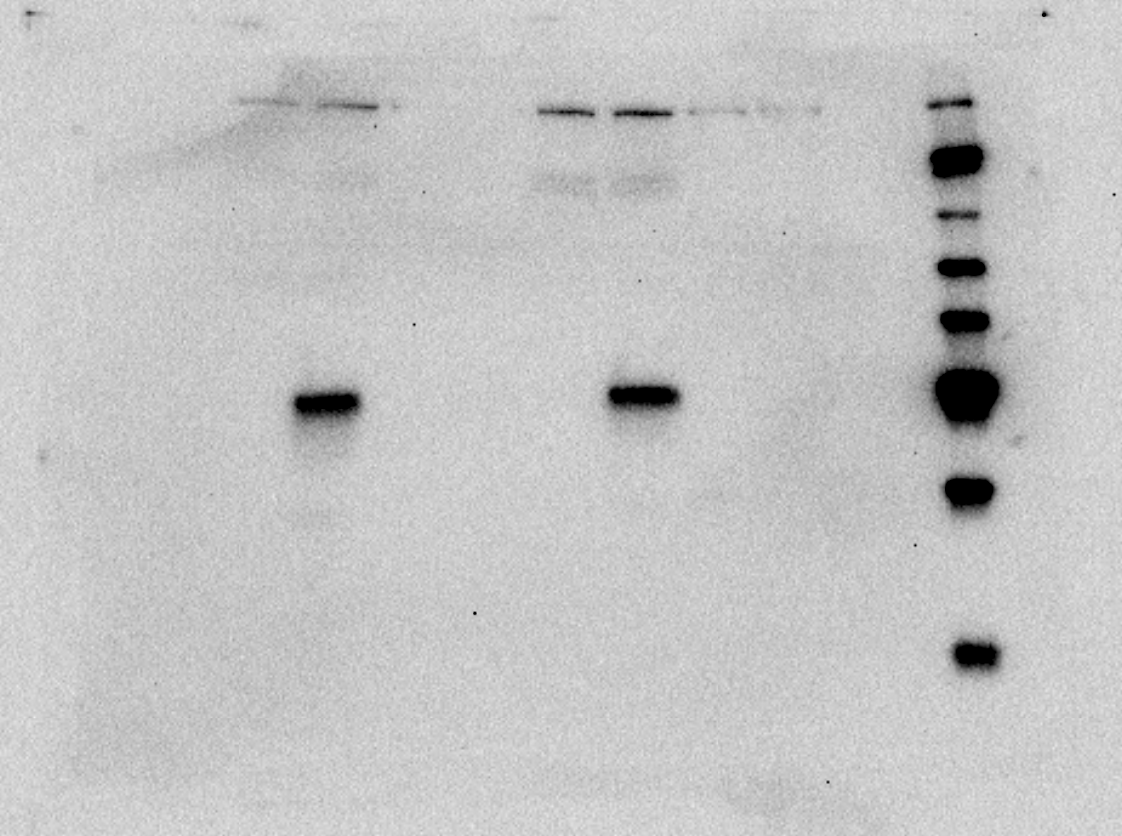

Supplement: Figure 1—figure supplement 2—source data 1. — Figures with the uncropped blots are clearly labeled with the relevant bands. [file elife-83189-fig1-figsupp2-data1.zip › Figure 1-figure supplement 2-source data 1/Fig. 1 Suppl 2G Poly-PG.tiff]

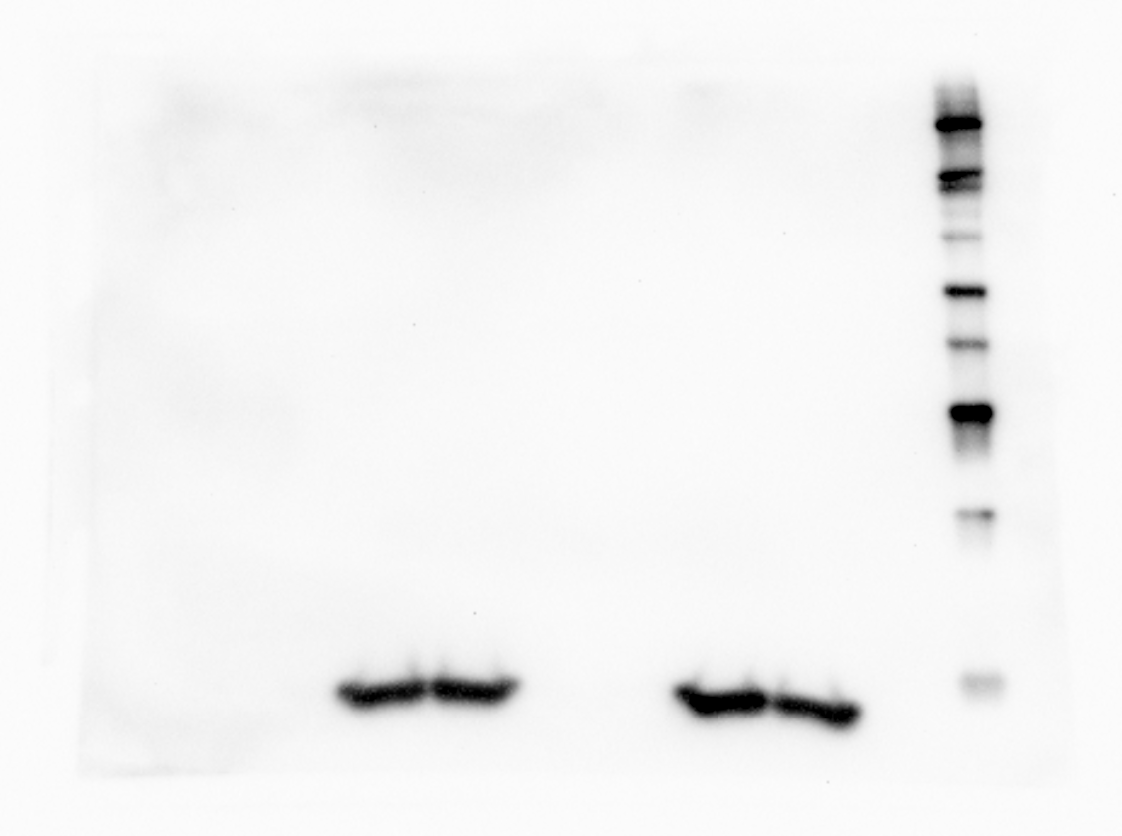

Supplement: Figure 1—figure supplement 2—source data 1. — Figures with the uncropped blots are clearly labeled with the relevant bands. [file elife-83189-fig1-figsupp2-data1.zip › Figure 1-figure supplement 2-source data 1/Fig. 1 Suppl 2B H3K4me2.tiff]

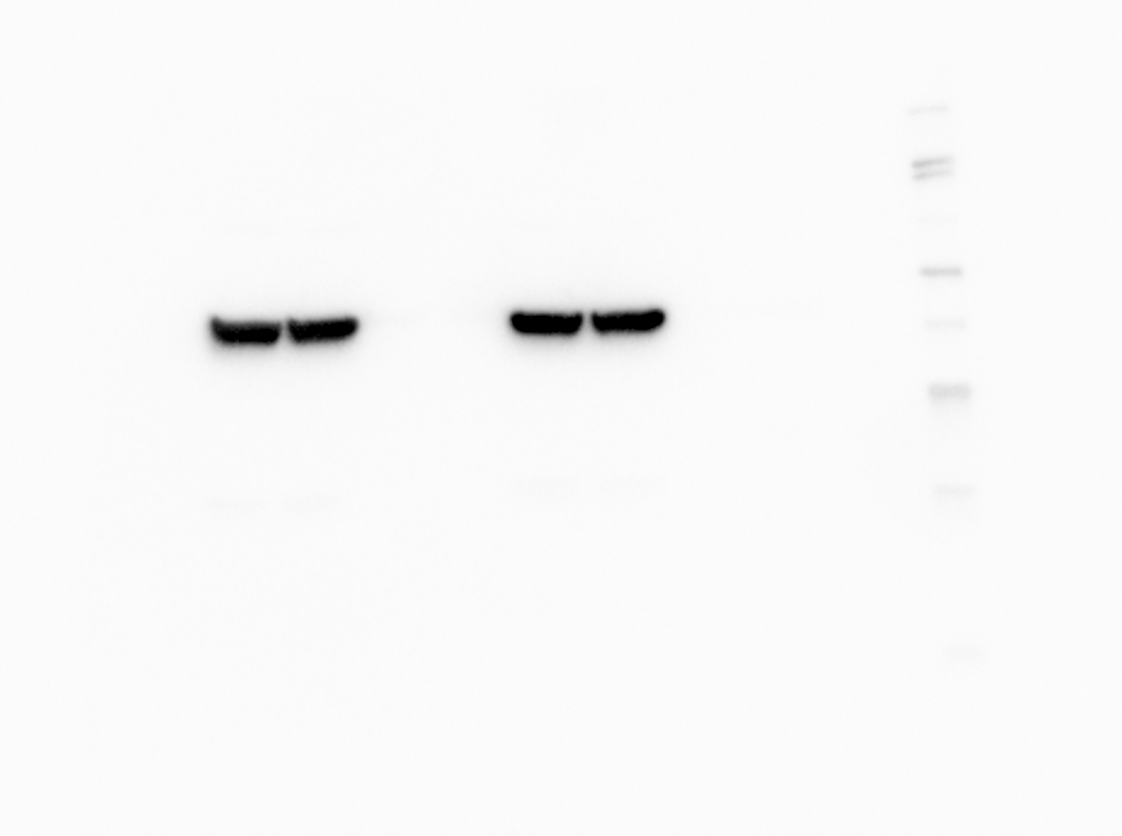

Supplement: Figure 1—figure supplement 2—source data 1. — Figures with the uncropped blots are clearly labeled with the relevant bands. [file elife-83189-fig1-figsupp2-data1.zip › Figure 1-figure supplement 2-source data 1/Fig. 1 Suppl 2G a-tubulin.tiff]

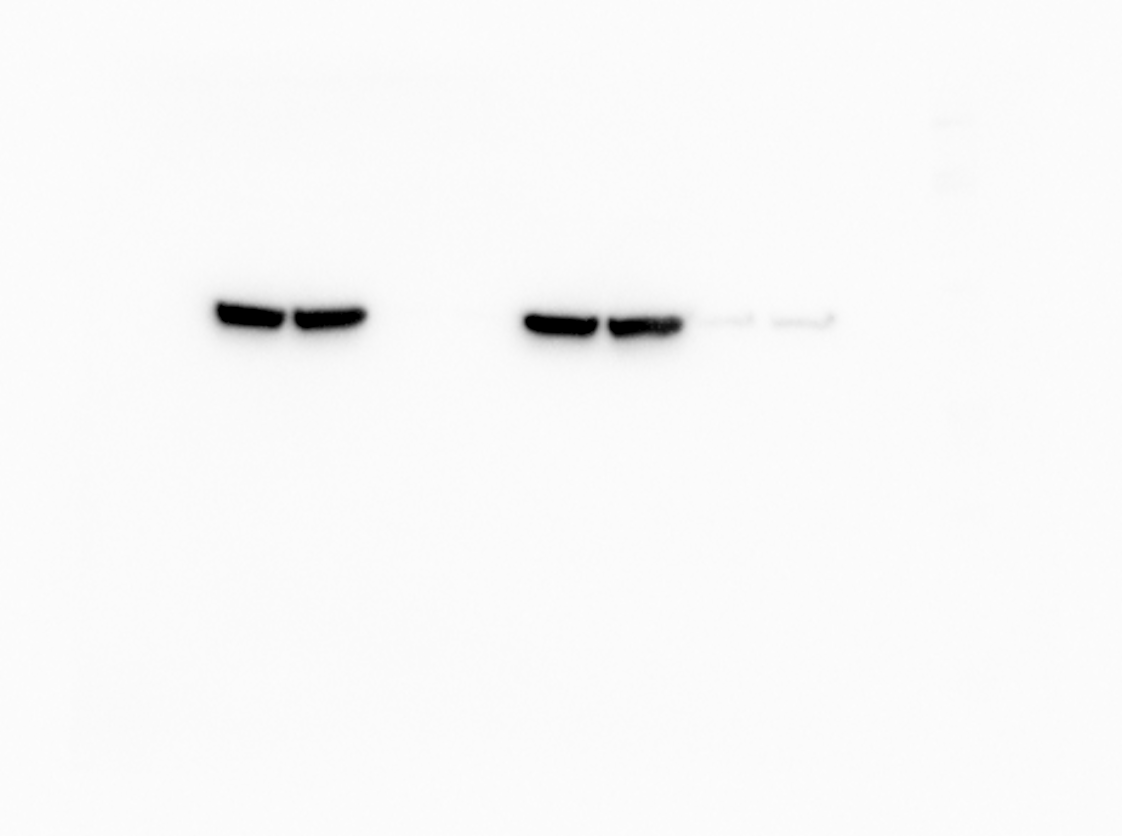

Supplement: Figure 1—figure supplement 2—source data 1. — Figures with the uncropped blots are clearly labeled with the relevant bands. [file elife-83189-fig1-figsupp2-data1.zip › Figure 1-figure supplement 2-source data 1/Fig. 1 Suppl 2B a-tubulin.tiff]

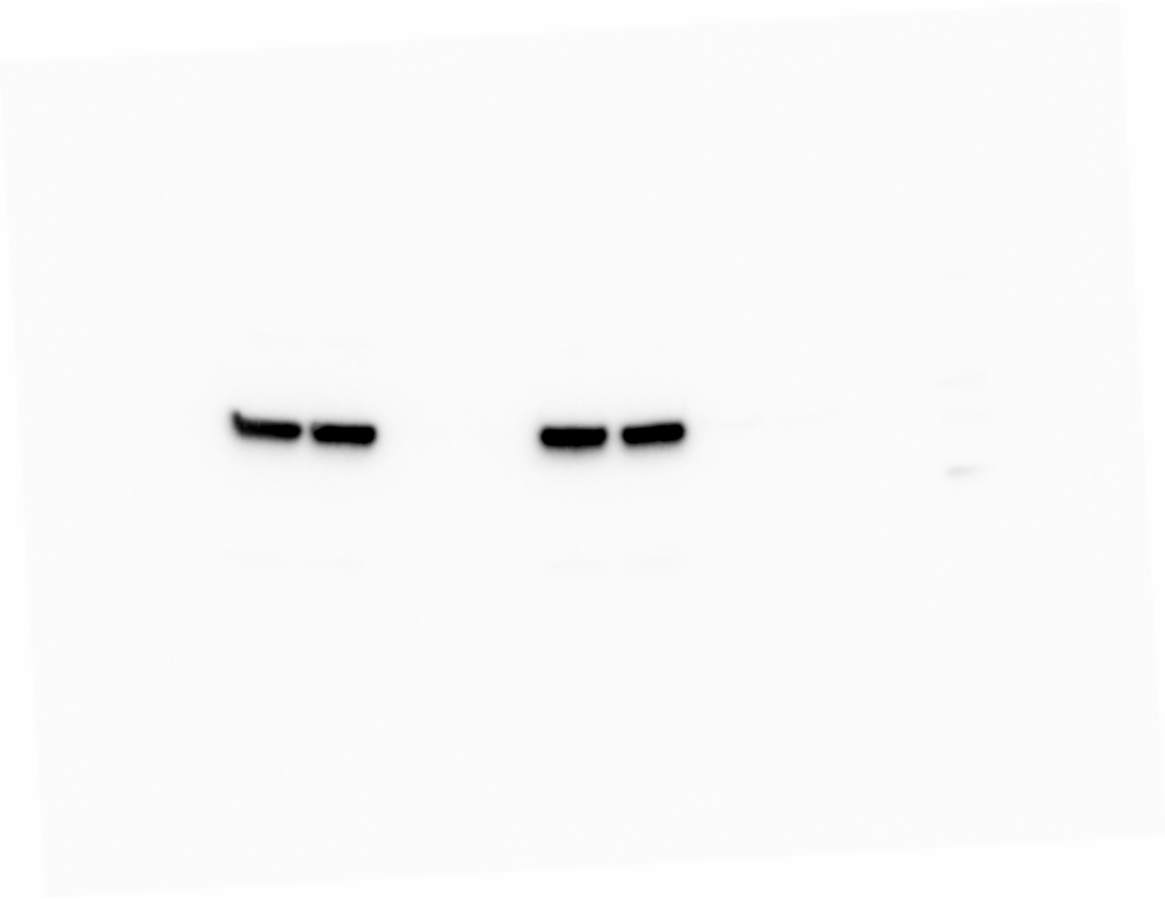

Supplement: Figure 1—figure supplement 2—source data 1. — Figures with the uncropped blots are clearly labeled with the relevant bands. [file elife-83189-fig1-figsupp2-data1.zip › Figure 1-figure supplement 2-source data 1/Fig. 1 Suppl 2C a-tubulin.tiff]

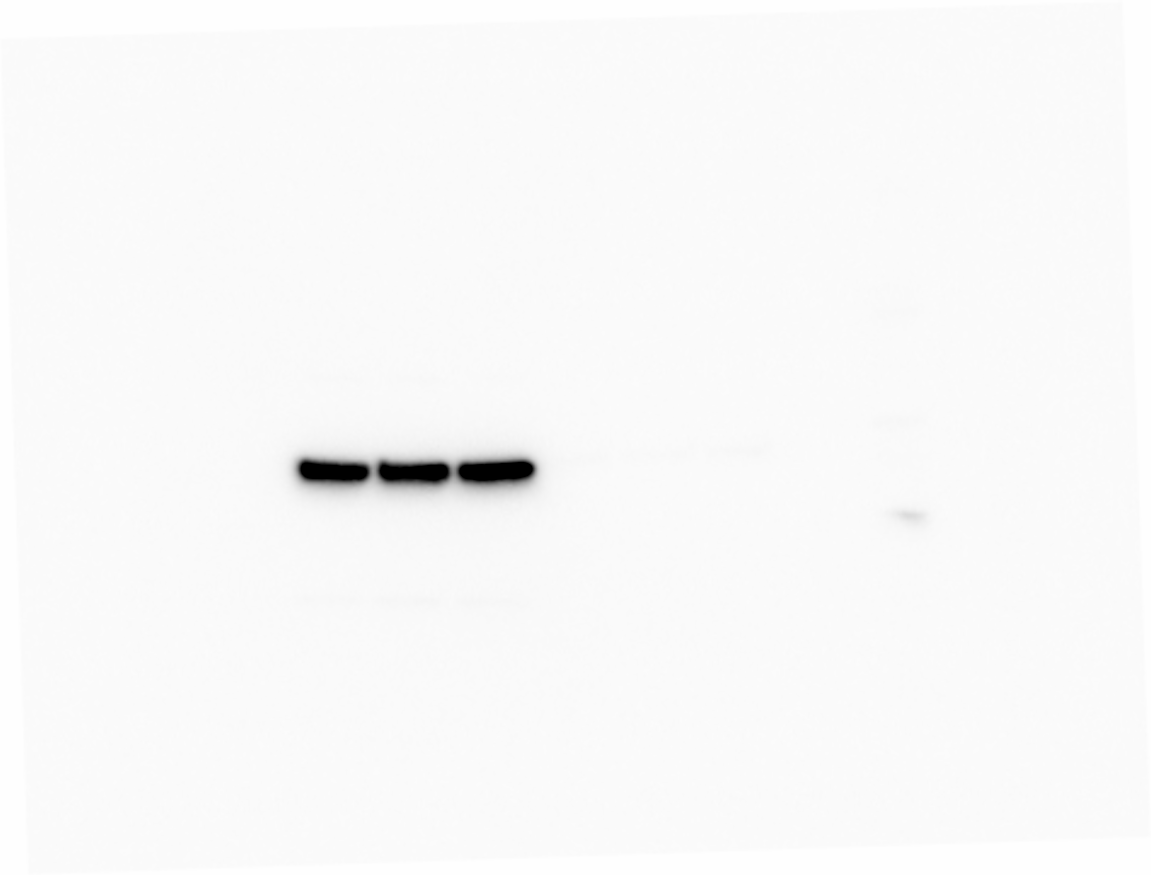

Supplement: Figure 1—figure supplement 3—source data 1. — Figures with the uncropped blots are clearly labeled with the relevant bands. [file elife-83189-fig1-figsupp3-data1.zip › Figure 1-figure supplement 3-source data 1/Fig. 1 Suppl 3C a-tubulin.tiff]

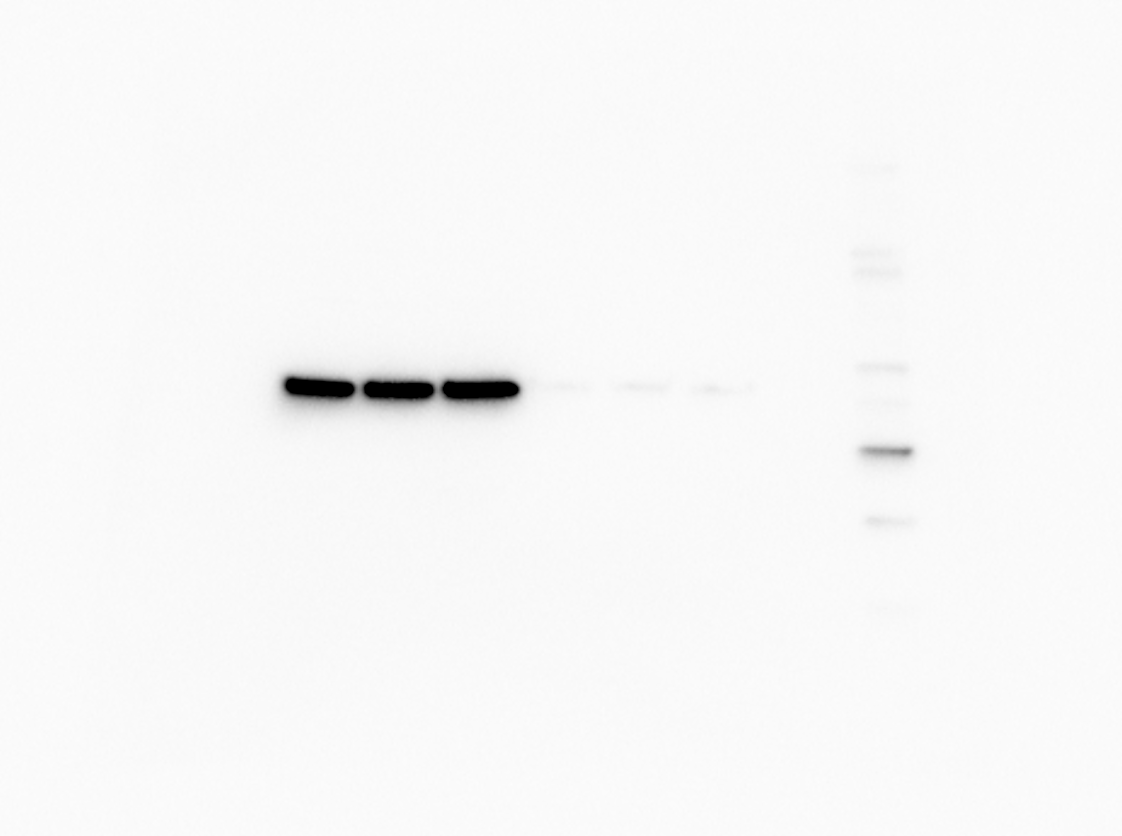

Supplement: Figure 1—figure supplement 3—source data 1. — Figures with the uncropped blots are clearly labeled with the relevant bands. [file elife-83189-fig1-figsupp3-data1.zip › Figure 1-figure supplement 3-source data 1/Fig. 1 Suppl 3B a-tubulin.tiff]

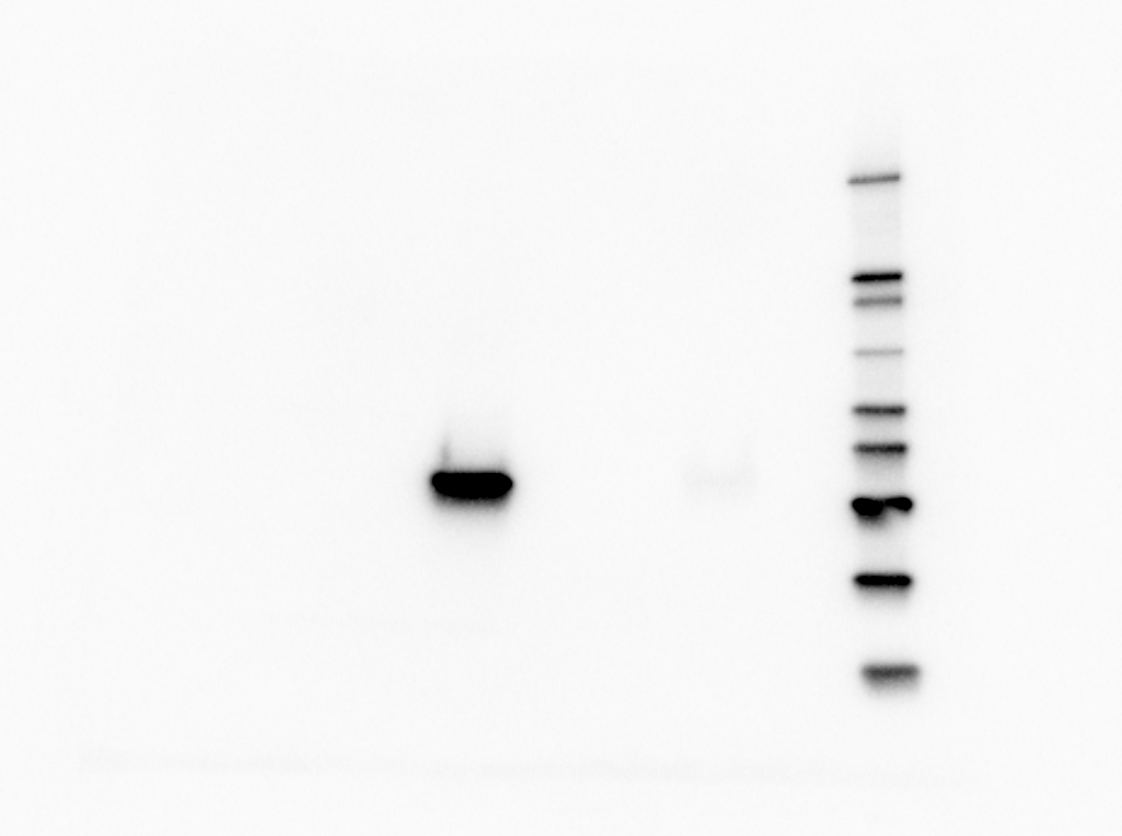

Supplement: Figure 1—figure supplement 3—source data 1. — Figures with the uncropped blots are clearly labeled with the relevant bands. [file elife-83189-fig1-figsupp3-data1.zip › Figure 1-figure supplement 3-source data 1/Fig. 1 Suppl 3C Poly-PA.tiff]

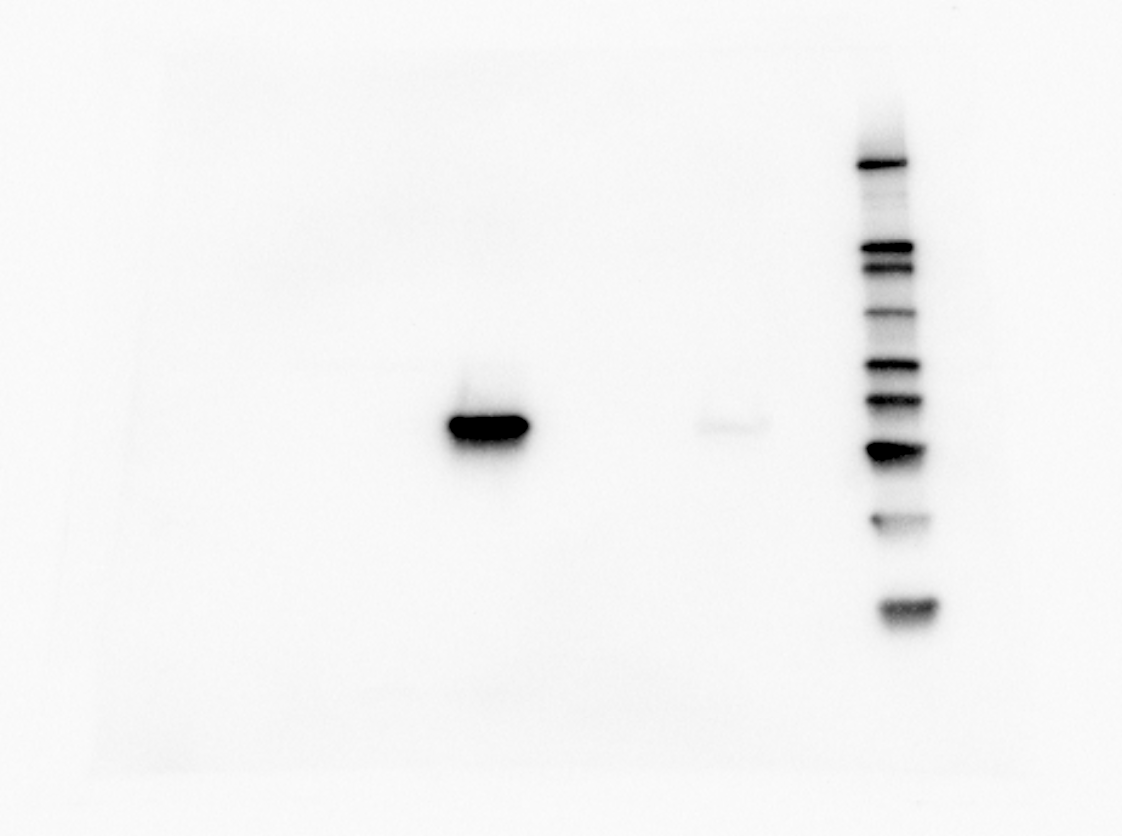

Supplement: Figure 1—figure supplement 3—source data 1. — Figures with the uncropped blots are clearly labeled with the relevant bands. [file elife-83189-fig1-figsupp3-data1.zip › Figure 1-figure supplement 3-source data 1/Fig. 1 Suppl 3B Poly-PA.tiff]

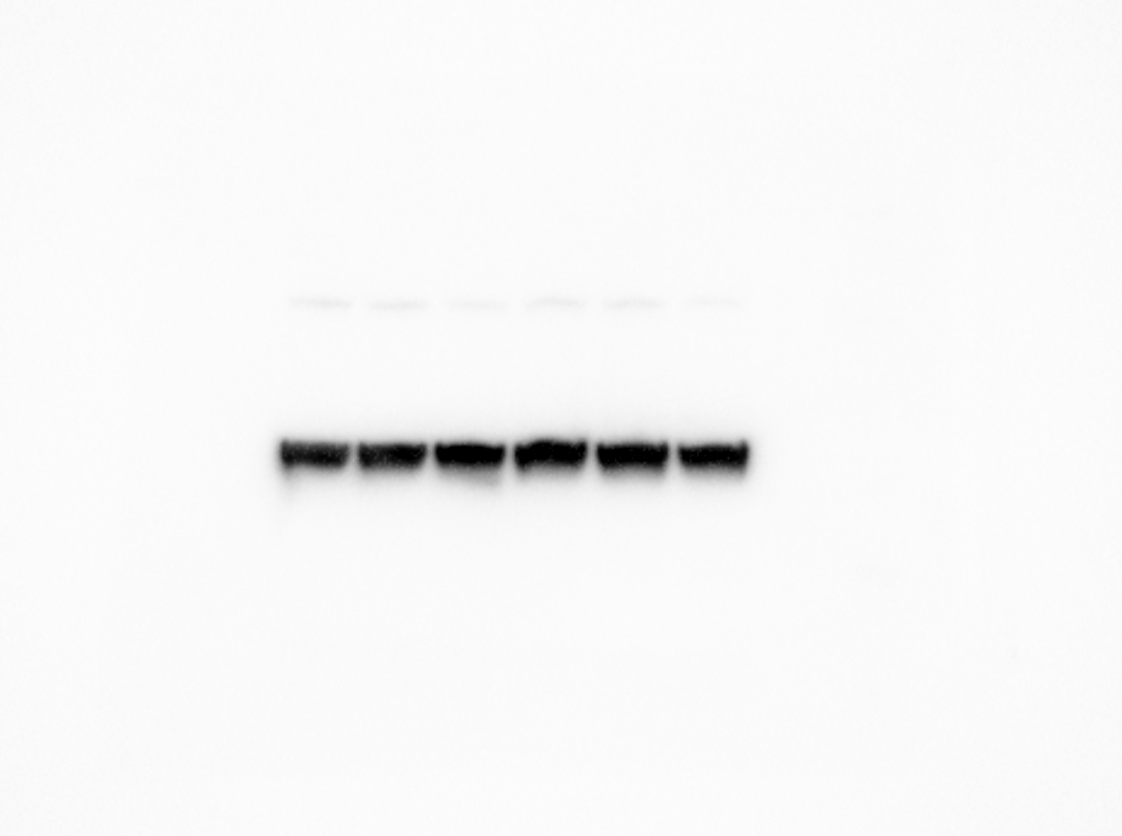

Supplement: Figure 2—source data 1. — Figures with the uncropped blots are clearly labeled with the relevant bands. [file elife-83189-fig2-data1.zip › Figure 2-source data 1/Fig. 2B a-tubulin.tif]

Figure 2B

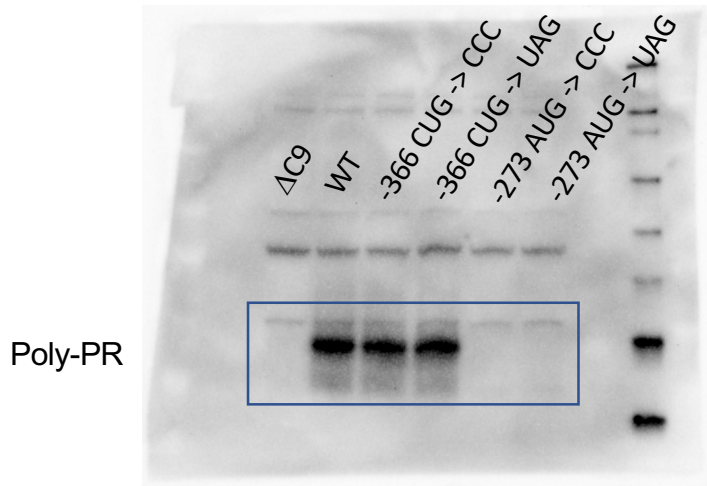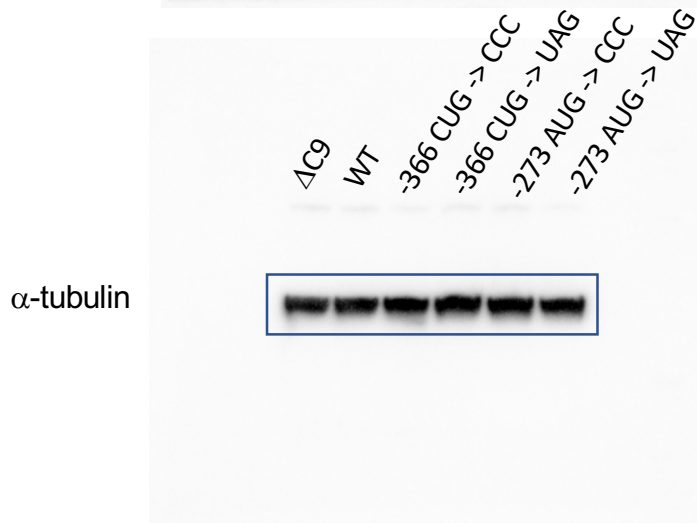

Figure 2D

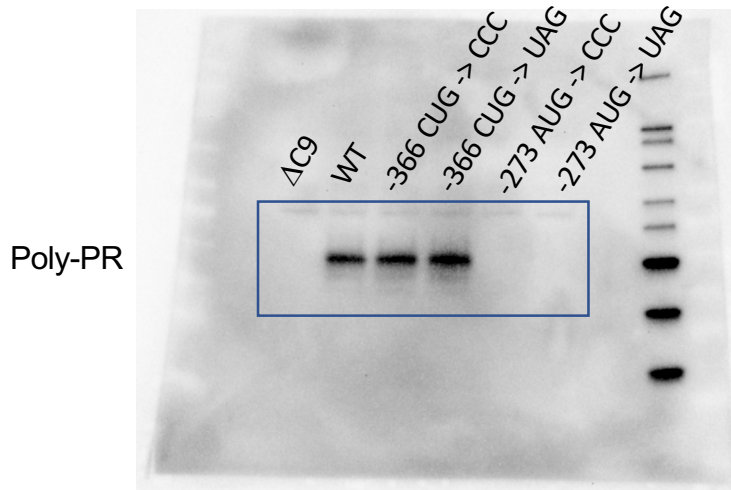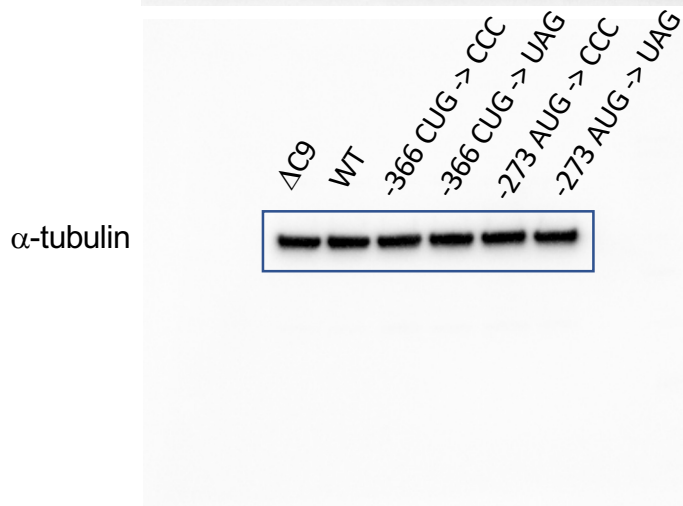

Supplement: Figure 2—source data 1. — Figures with the uncropped blots are clearly labeled with the relevant bands. [file elife-83189-fig2-data1.zip › Figure 2-source data 1/Figure 2-source data.pdf]

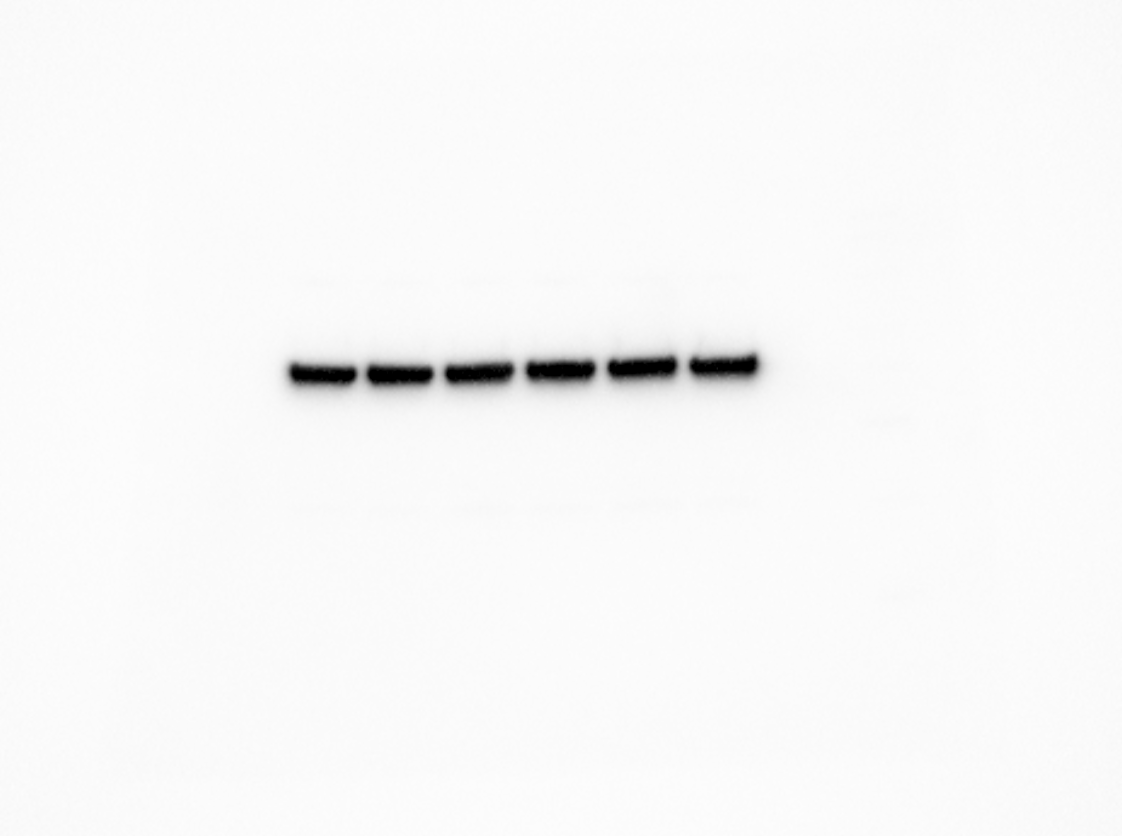

Supplement: Figure 2—source data 1. — Figures with the uncropped blots are clearly labeled with the relevant bands. [file elife-83189-fig2-data1.zip › Figure 2-source data 1/Fig. 2D a-tubulin.tif]

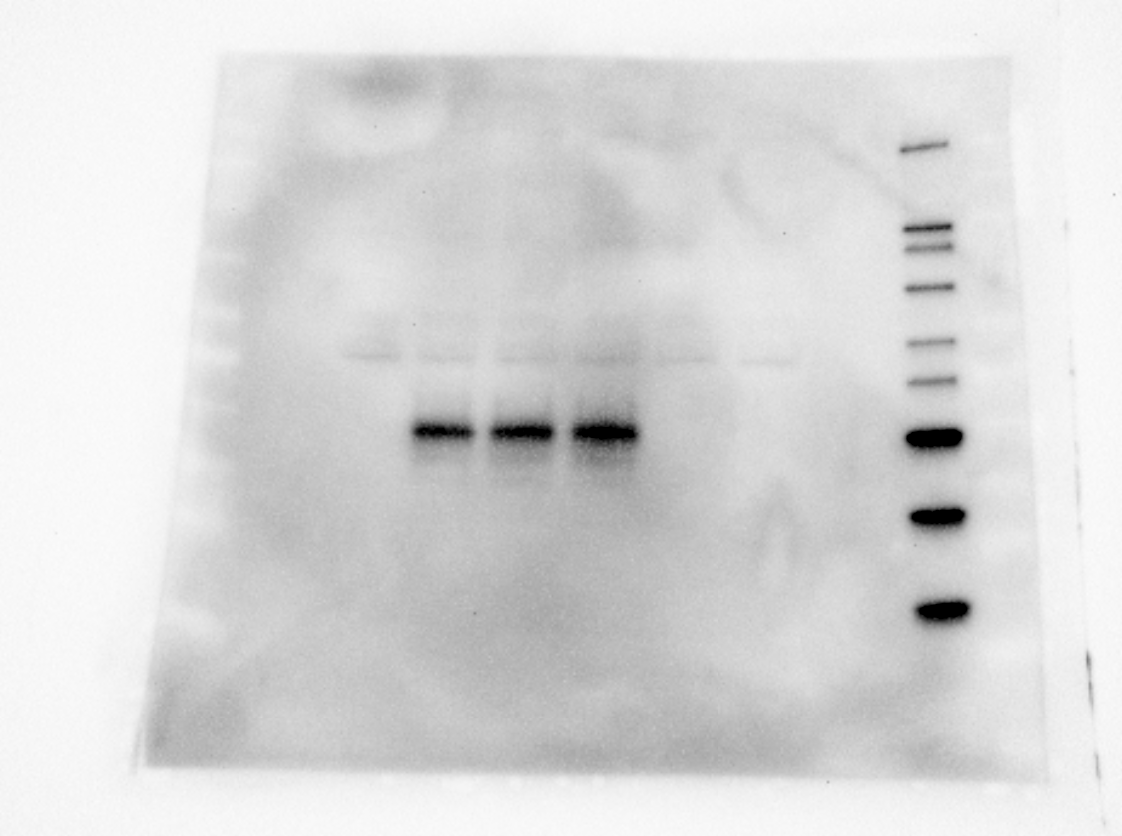

Supplement: Figure 2—source data 1. — Figures with the uncropped blots are clearly labeled with the relevant bands. [file elife-83189-fig2-data1.zip › Figure 2-source data 1/Fig. 2D Poly-PR.tif]

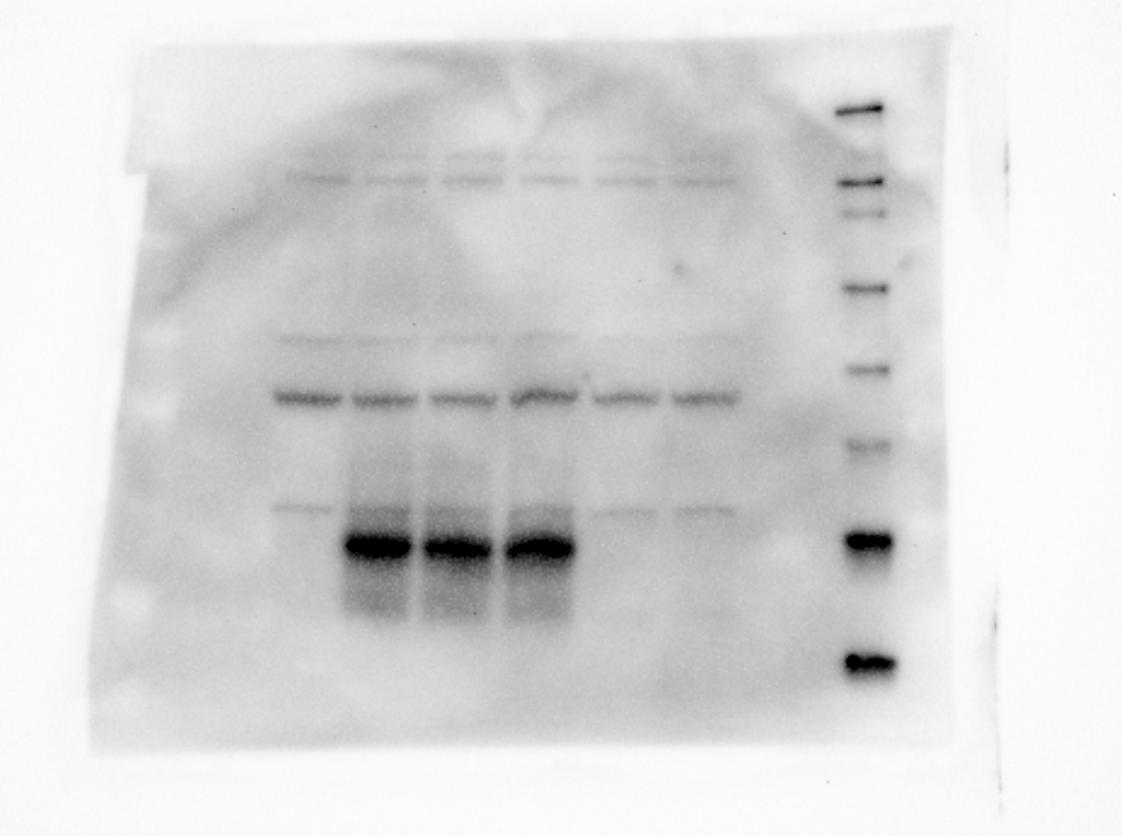

Supplement: Figure 2—source data 1. — Figures with the uncropped blots are clearly labeled with the relevant bands. [file elife-83189-fig2-data1.zip › Figure 2-source data 1/Fig. 2B Poly-PR.tif]

Figure 3B, HEK293 cells

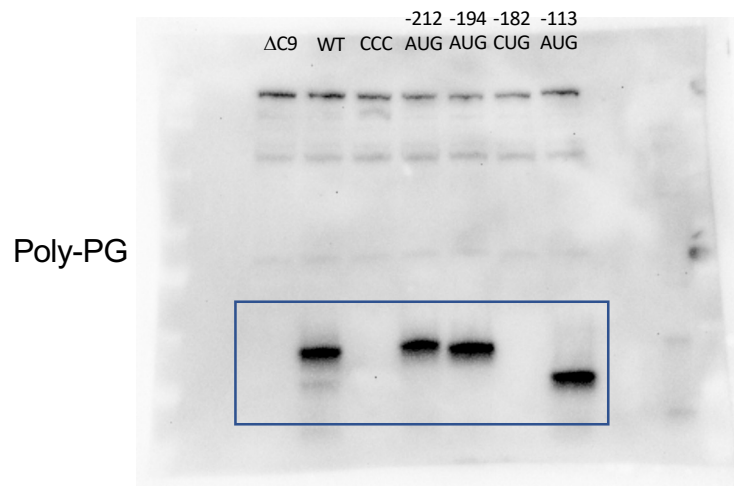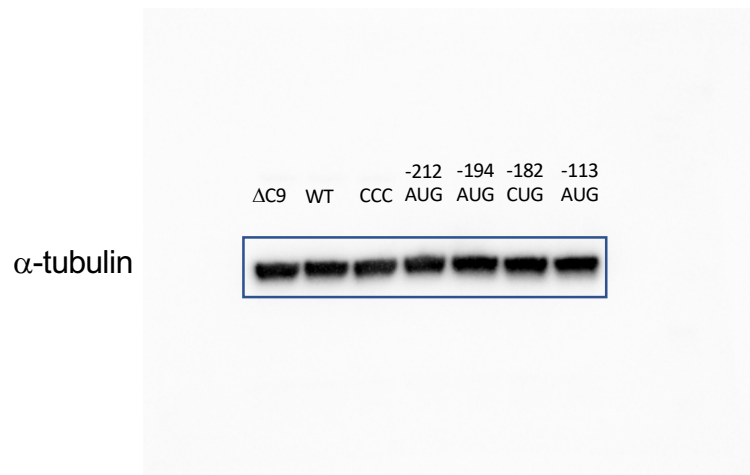

Figure 3B, NSC34 cells

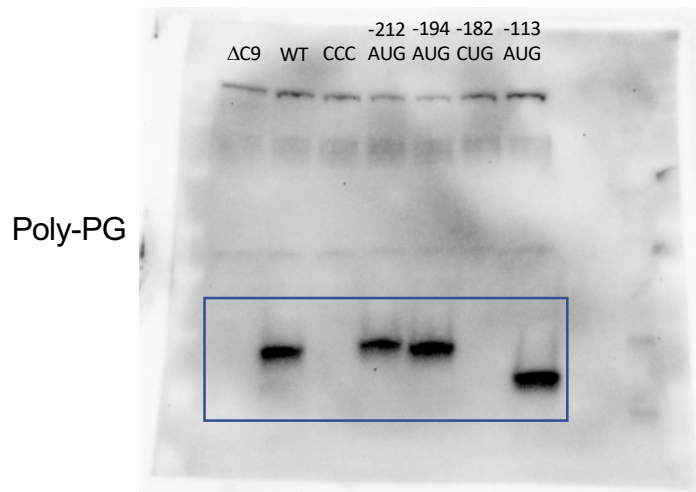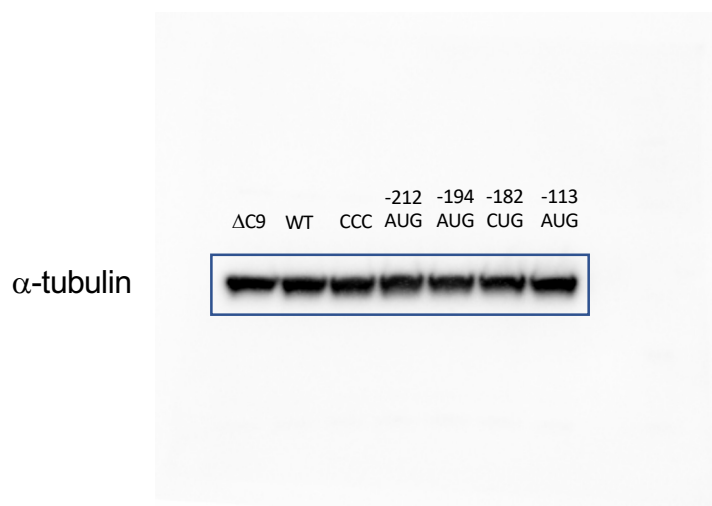

Supplement: Figure 3—source data 1. — Figures with the uncropped blots are clearly labeled with the relevant bands. [file elife-83189-fig3-data1.zip › Figure 3-source data 1/Figure 3-source data labelled.pdf]

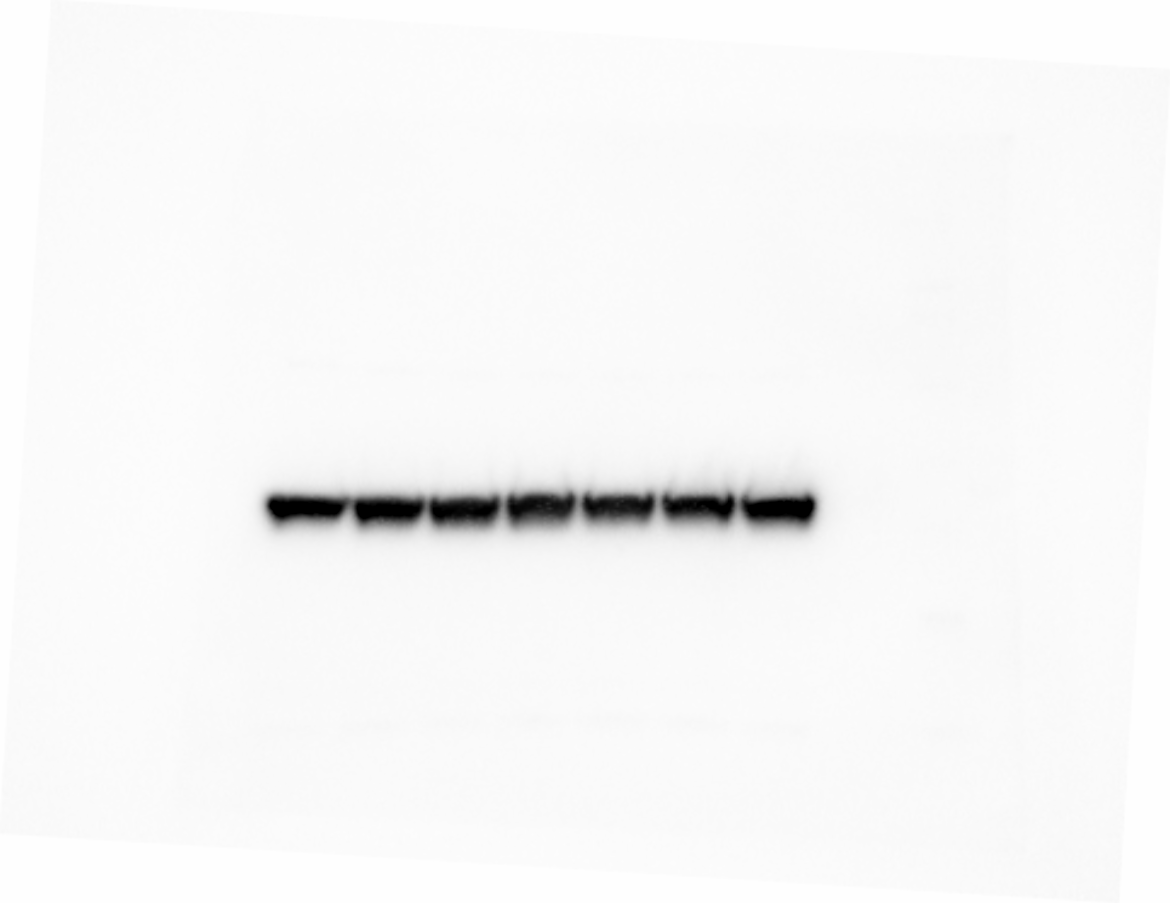

Supplement: Figure 3—source data 1. — Figures with the uncropped blots are clearly labeled with the relevant bands. [file elife-83189-fig3-data1.zip › Figure 3-source data 1/Fig. 3B NSC34 a-tubulin.tif]

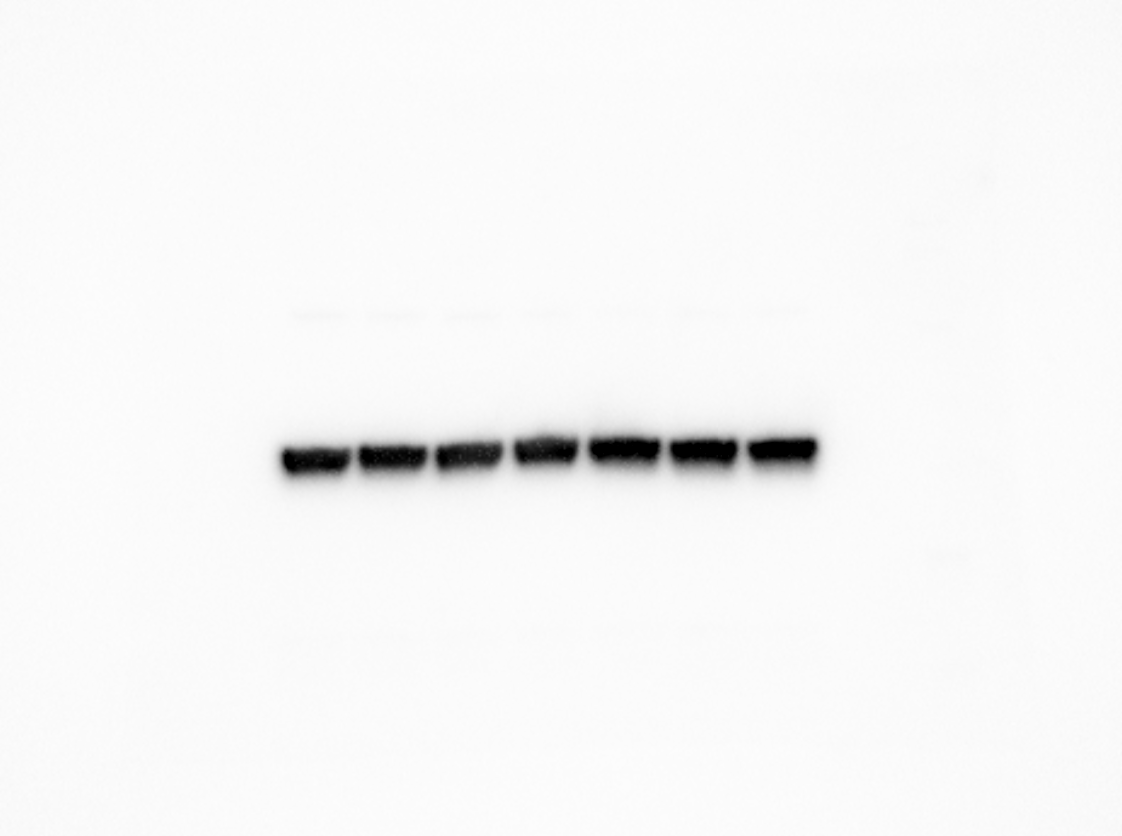

Supplement: Figure 3—source data 1. — Figures with the uncropped blots are clearly labeled with the relevant bands. [file elife-83189-fig3-data1.zip › Figure 3-source data 1/Fig. 3B HEK293 a-tubulin.tif]

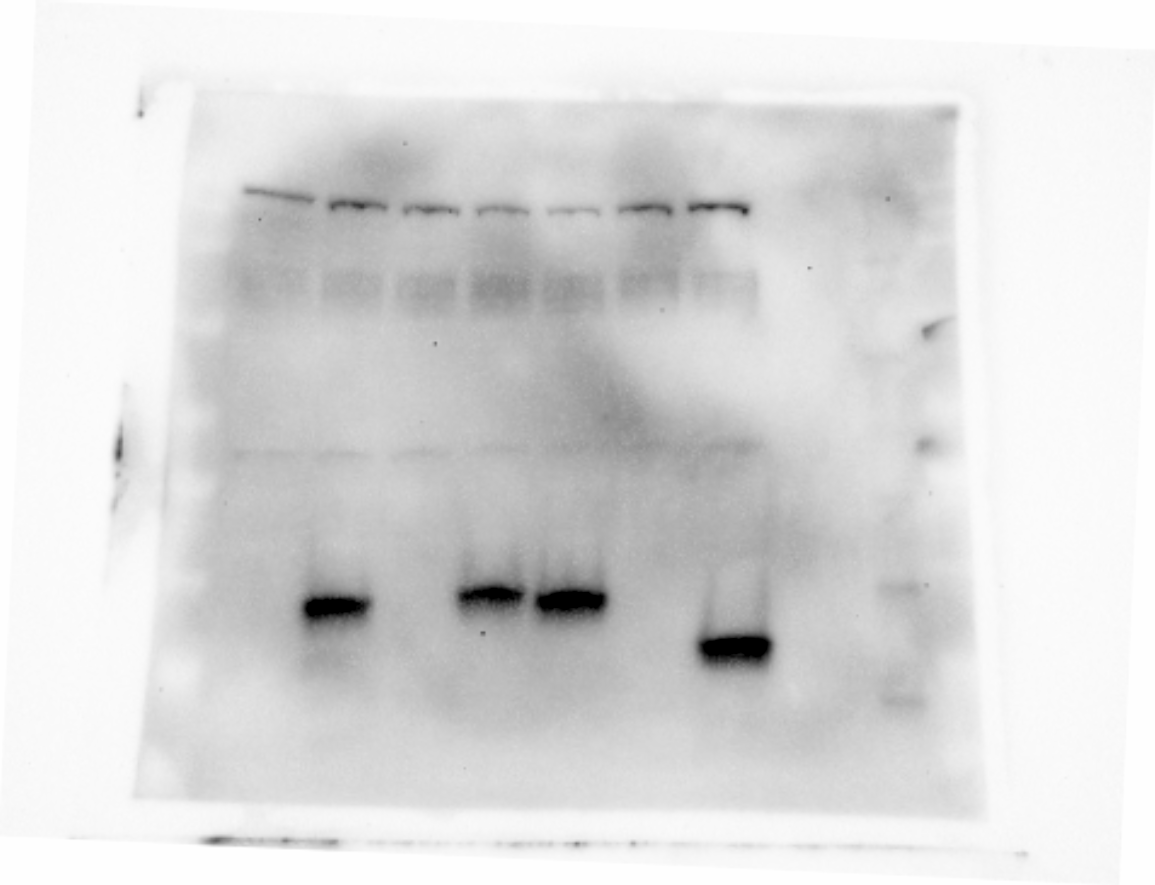

Supplement: Figure 3—source data 1. — Figures with the uncropped blots are clearly labeled with the relevant bands. [file elife-83189-fig3-data1.zip › Figure 3-source data 1/Fig. 3B NSC34 Poly-PG.tif]

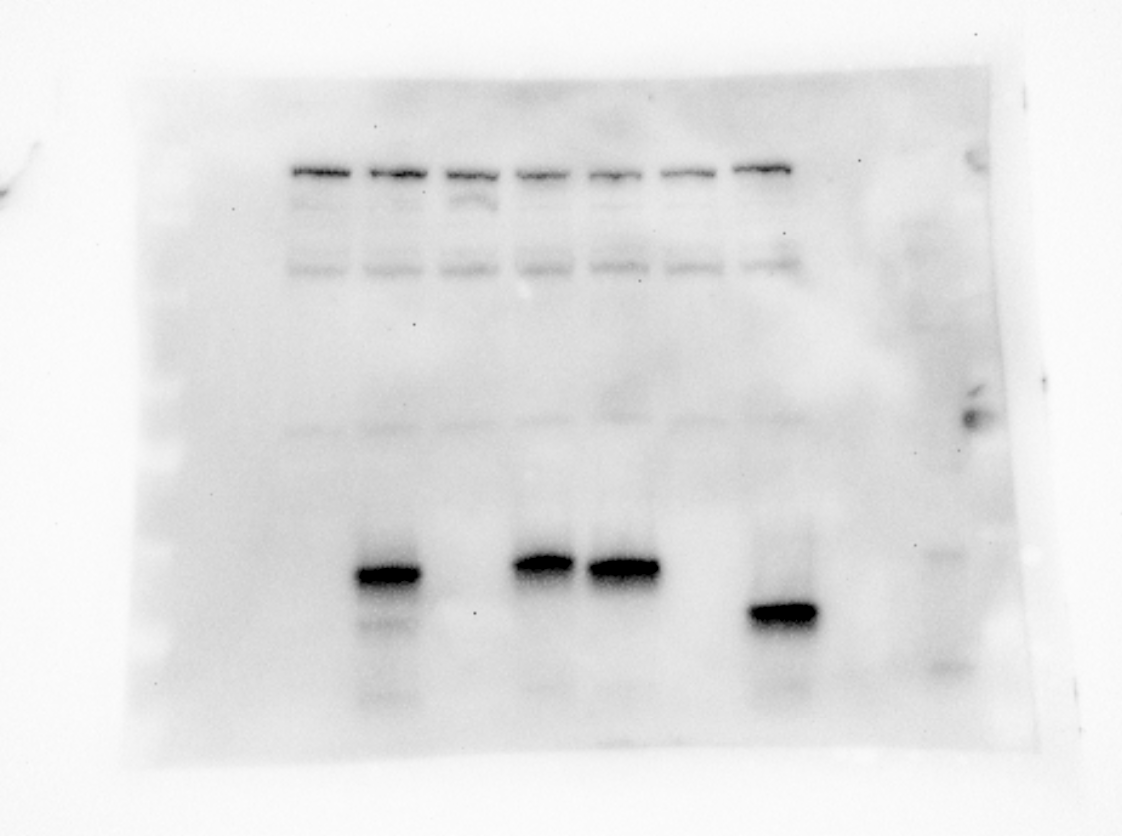

Supplement: Figure 3—source data 1. — Figures with the uncropped blots are clearly labeled with the relevant bands. [file elife-83189-fig3-data1.zip › Figure 3-source data 1/Fig. 3B HEK293 Poly-PG.tif]

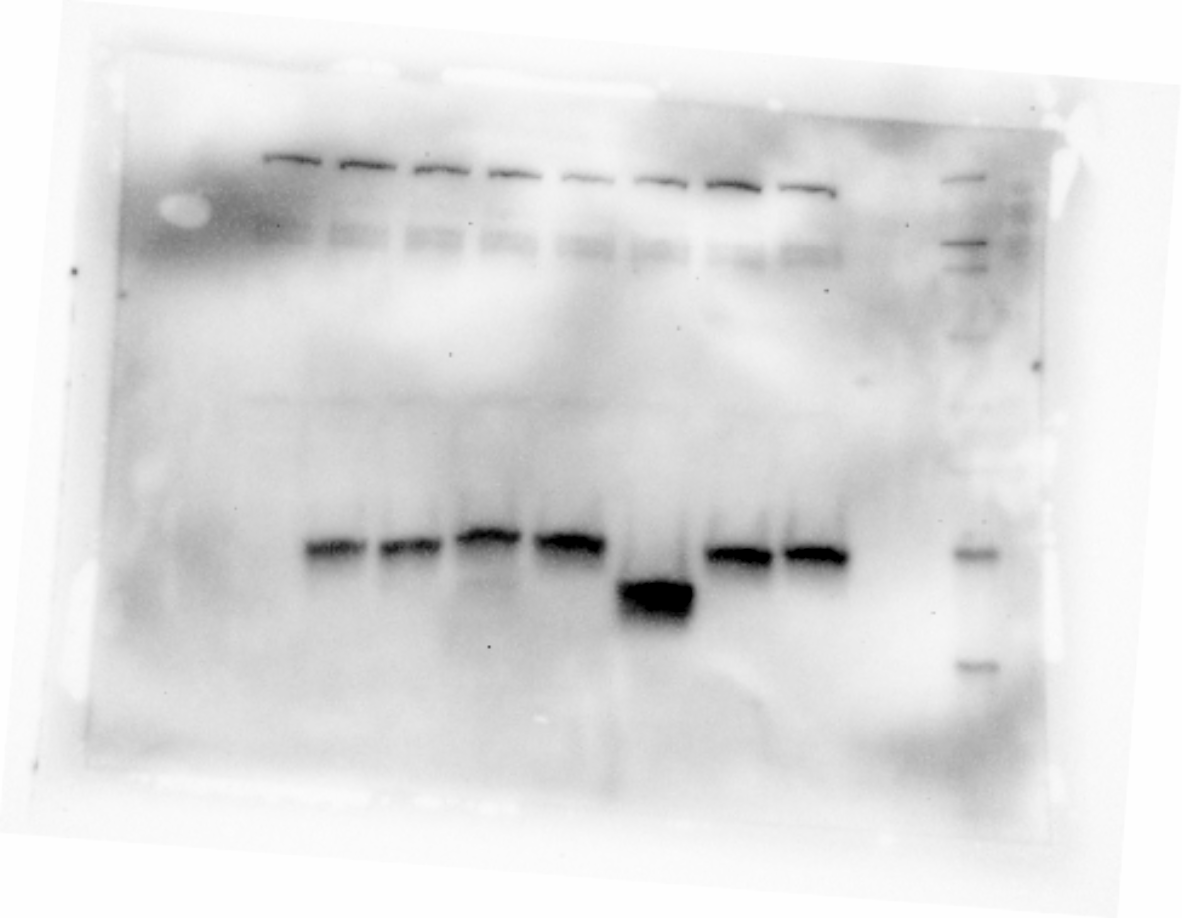

Supplement: Figure 4—source data 1. — Figures with the uncropped blots are clearly labeled with the relevant bands. [file elife-83189-fig4-data1.zip › Figure 4-source data 1/Fig. 4B NSC34 Poly-PG.tif]

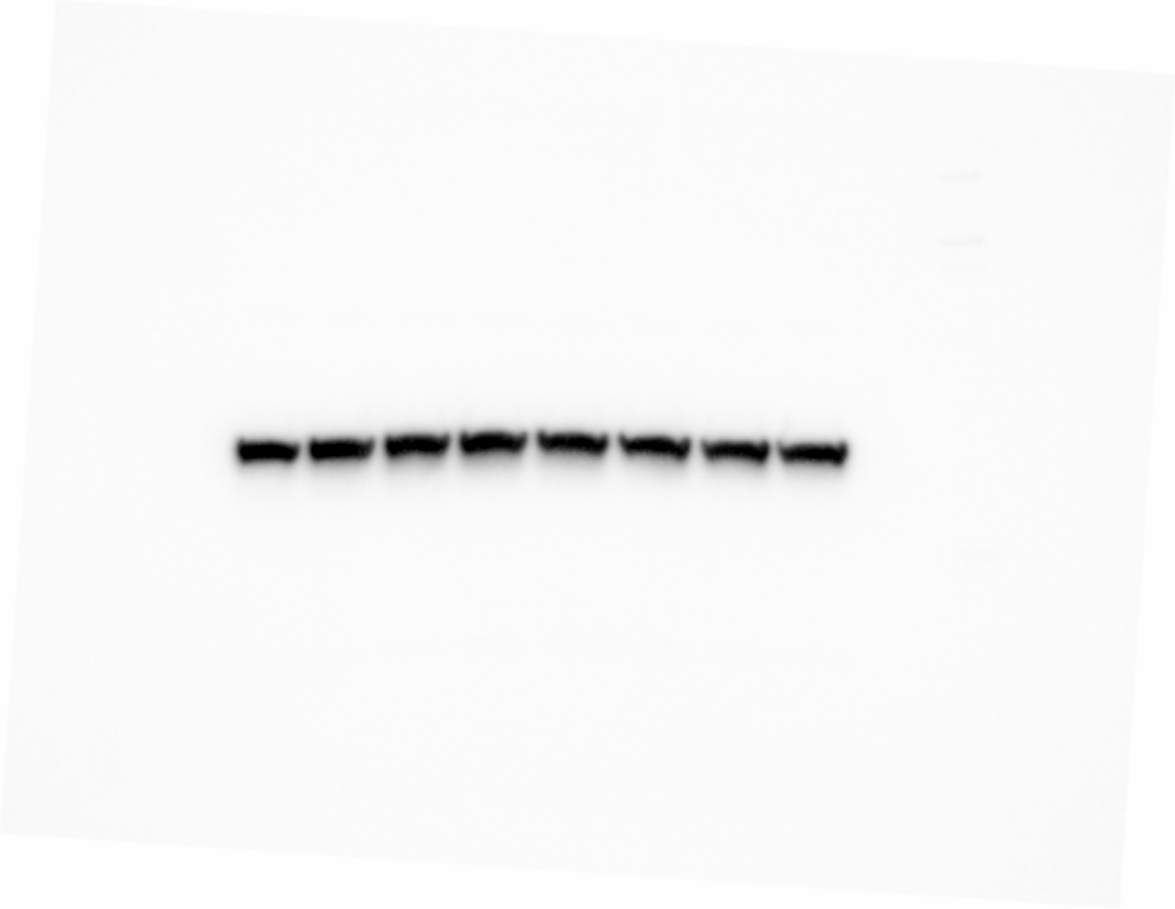

Supplement: Figure 4—source data 1. — Figures with the uncropped blots are clearly labeled with the relevant bands. [file elife-83189-fig4-data1.zip › Figure 4-source data 1/Fig. 4B NSC34 a-tubulin.tif]

Figure 4B, HEK293 cells

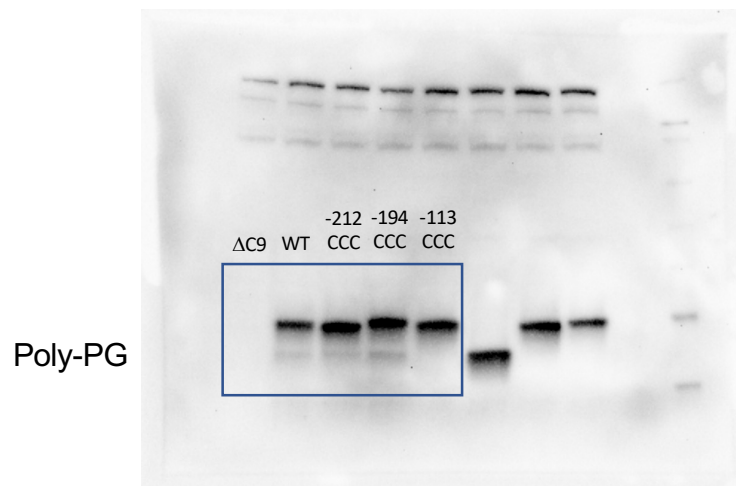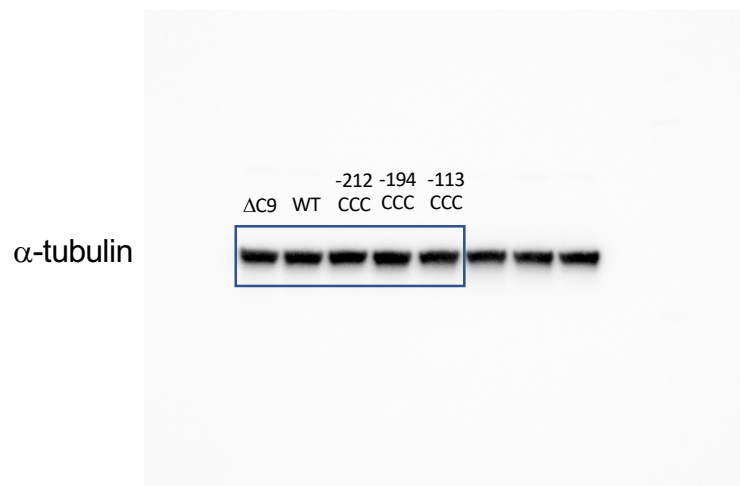

Figure 4B, NSC34 cells

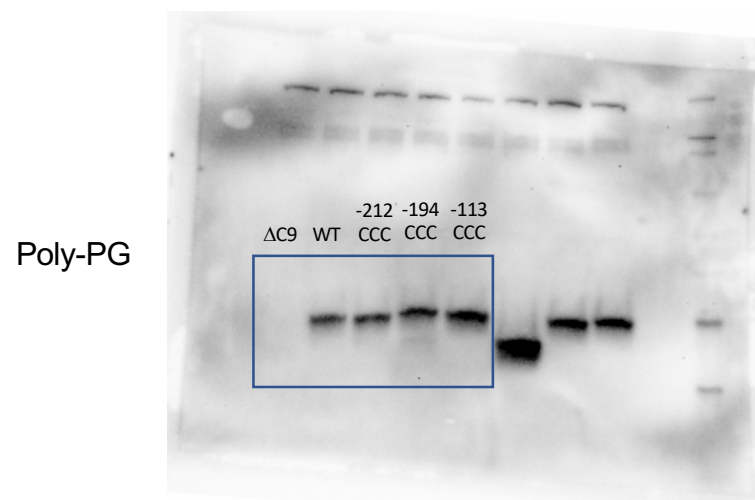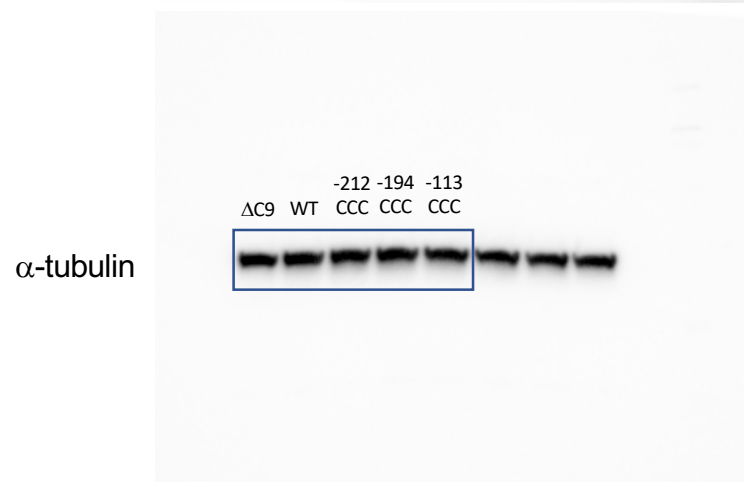

Supplement: Figure 4—source data 1. — Figures with the uncropped blots are clearly labeled with the relevant bands. [file elife-83189-fig4-data1.zip › Figure 4-source data 1/Figure 4-source data labelled.pdf]

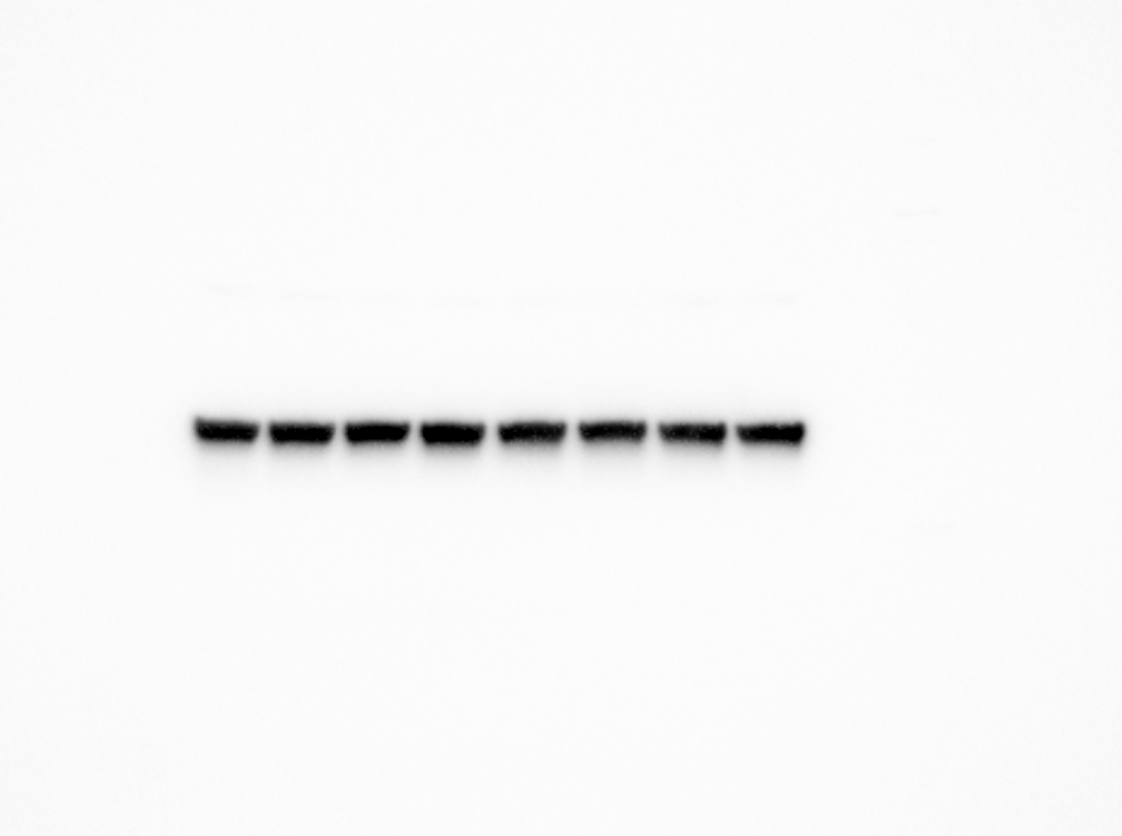

Supplement: Figure 4—source data 1. — Figures with the uncropped blots are clearly labeled with the relevant bands. [file elife-83189-fig4-data1.zip › Figure 4-source data 1/Fig. 4B HEK293 a-tubulin.tif]

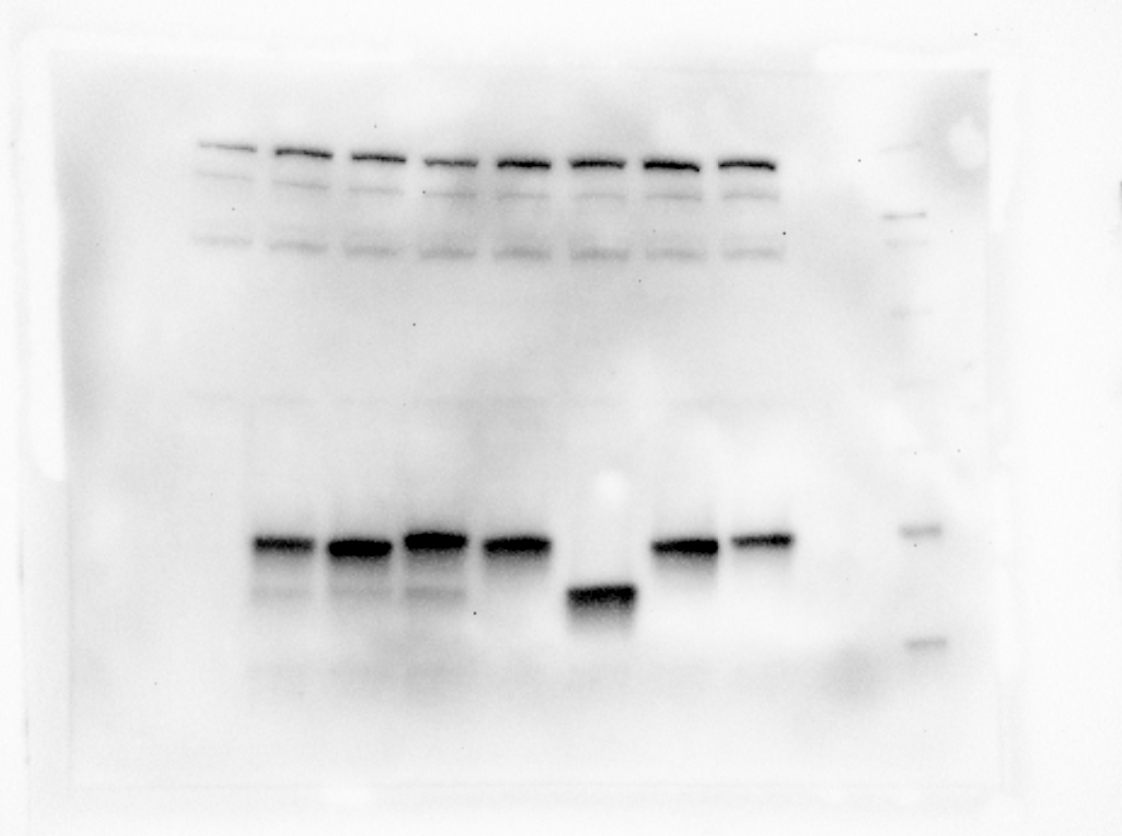

Supplement: Figure 4—source data 1. — Figures with the uncropped blots are clearly labeled with the relevant bands. [file elife-83189-fig4-data1.zip › Figure 4-source data 1/Fig. 4B HEK293 Poly-PG.tif]

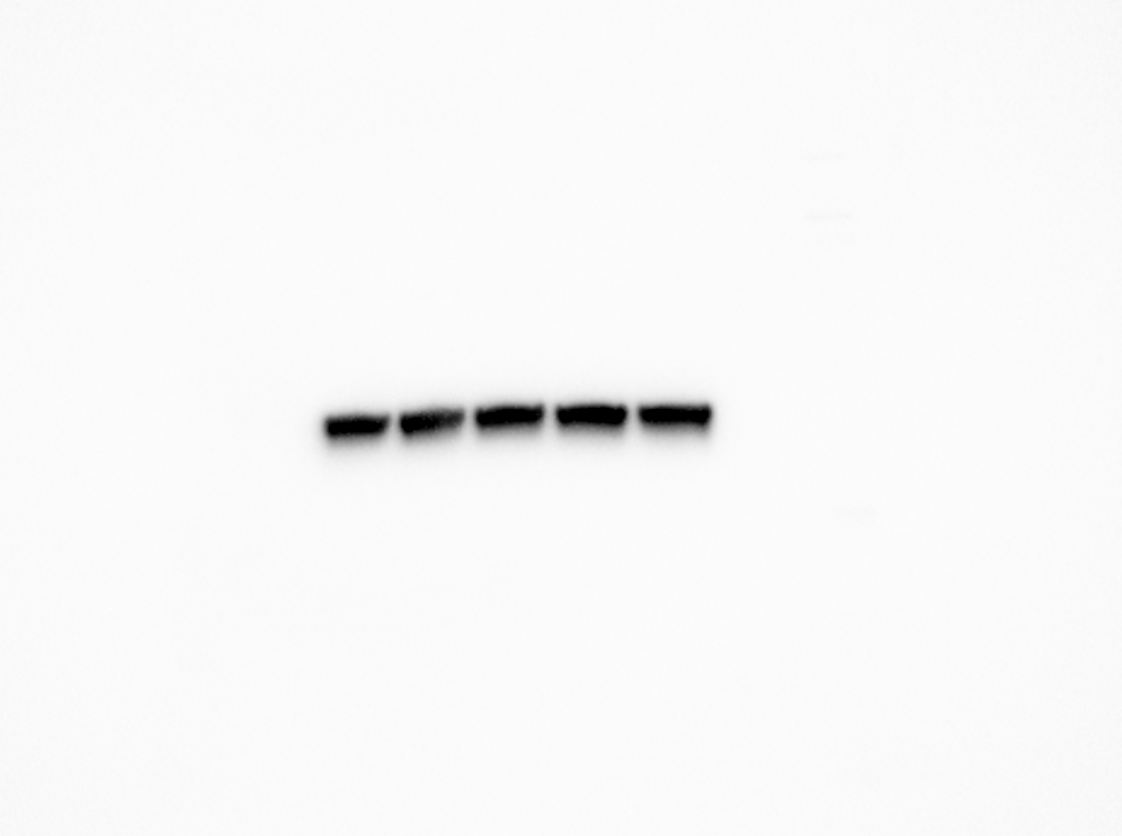

Supplement: Figure 5—source data 1. — Figures with the uncropped blots are clearly labeled with the relevant bands. [file elife-83189-fig5-data1.zip › Figure 5-source data 1/Fig. 5B HEK293 a-tubulin.tif]

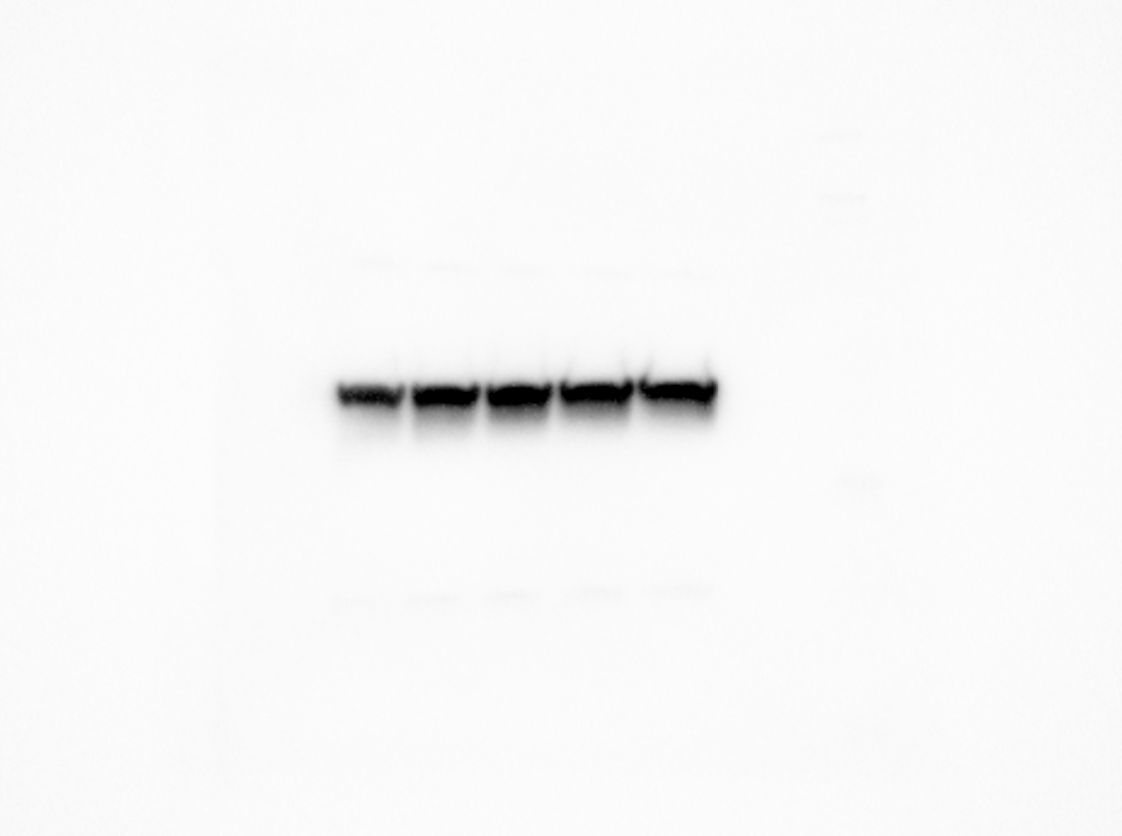

Supplement: Figure 5—source data 1. — Figures with the uncropped blots are clearly labeled with the relevant bands. [file elife-83189-fig5-data1.zip › Figure 5-source data 1/Fig. 5B NSC34 a-tubulin.tif]

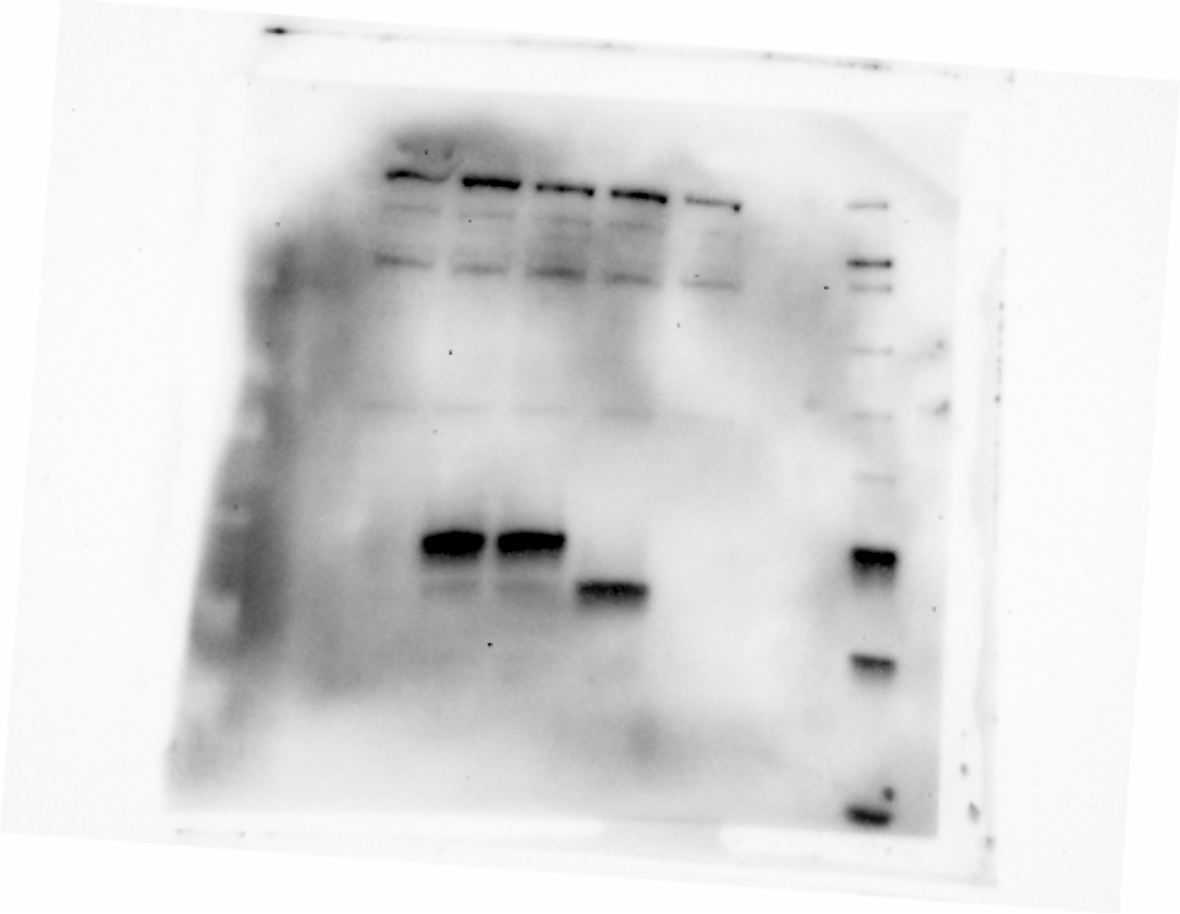

Supplement: Figure 5—source data 1. — Figures with the uncropped blots are clearly labeled with the relevant bands. [file elife-83189-fig5-data1.zip › Figure 5-source data 1/Fig. 5B HEK293 Poly-PG.tif]

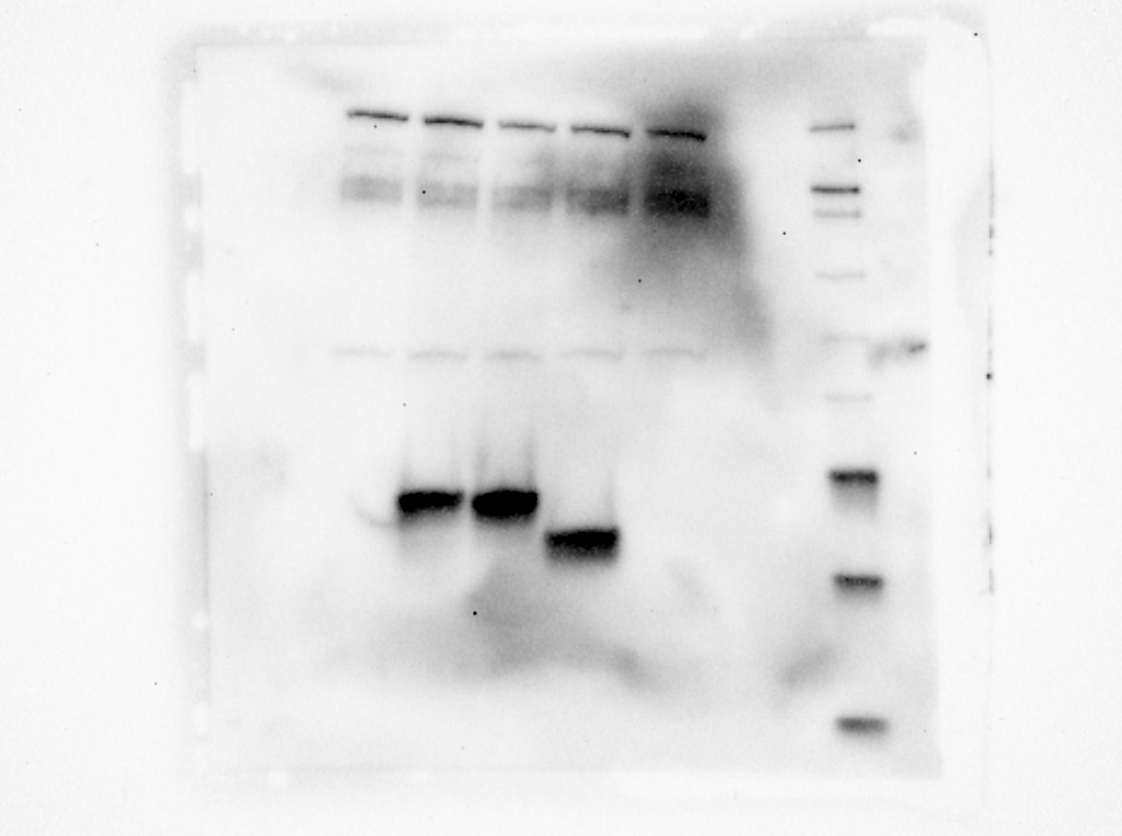

Supplement: Figure 5—source data 1. — Figures with the uncropped blots are clearly labeled with the relevant bands. [file elife-83189-fig5-data1.zip › Figure 5-source data 1/Fig. 5B NSC34 Poly-PG.tif]

Figure 5B, HEK293 cells

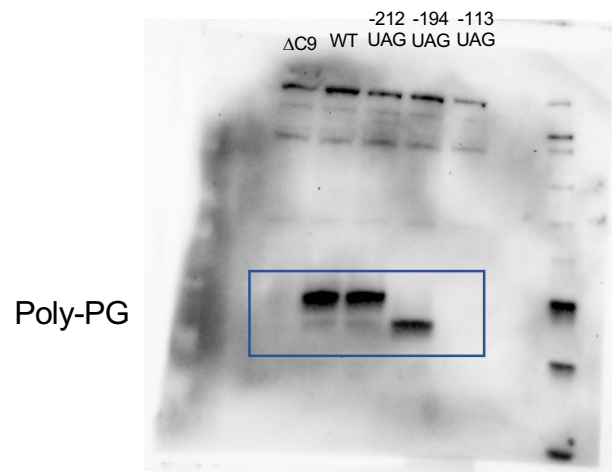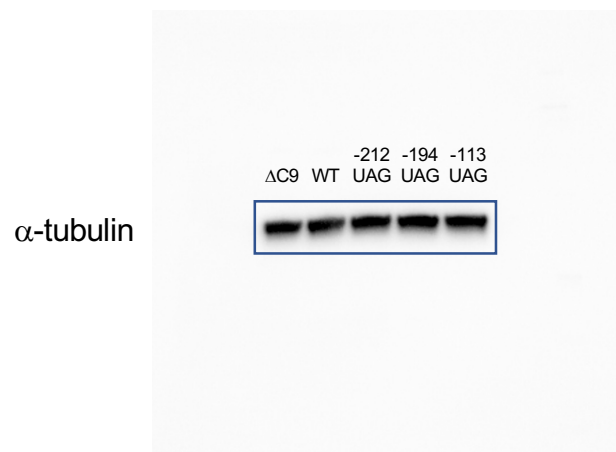

Figure 5B, NSC34 cells

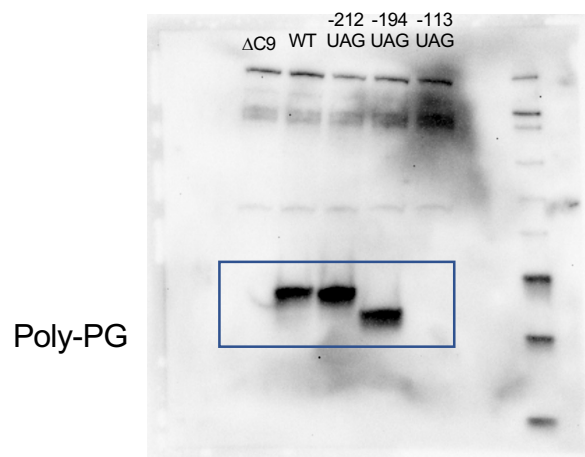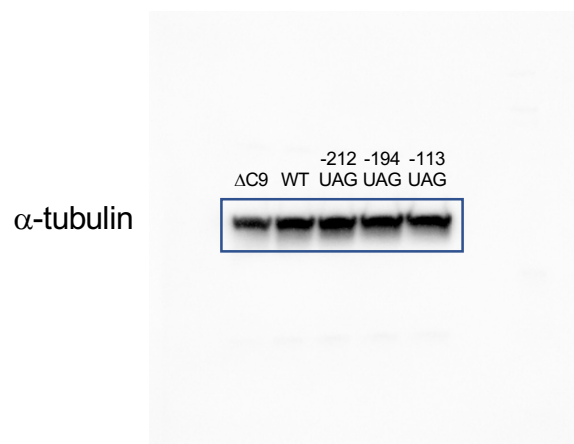

Supplement: Figure 5—source data 1. — Figures with the uncropped blots are clearly labeled with the relevant bands. [file elife-83189-fig5-data1.zip › Figure 5-source data 1/Figure 5-source data labelled.pdf]

Figure 6C

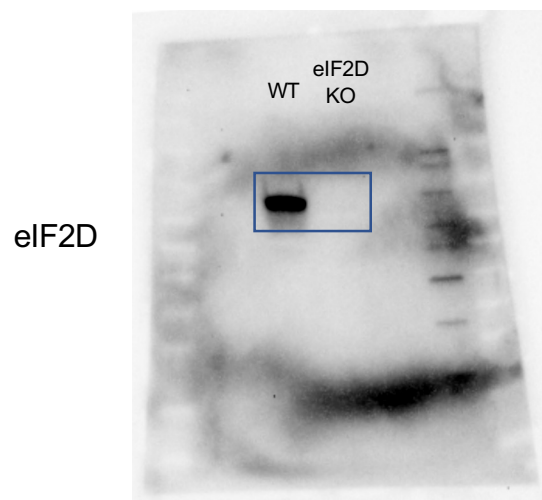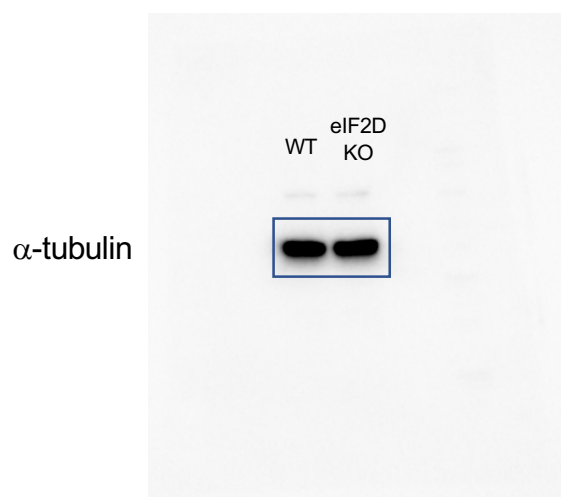

Supplement: Figure 6—source data 1. — Figures with the uncropped blots are clearly labeled with the relevant bands. [file elife-83189-fig6-data1.zip › Figure 6-source data 1/Figure 6-source data labelled.pdf]

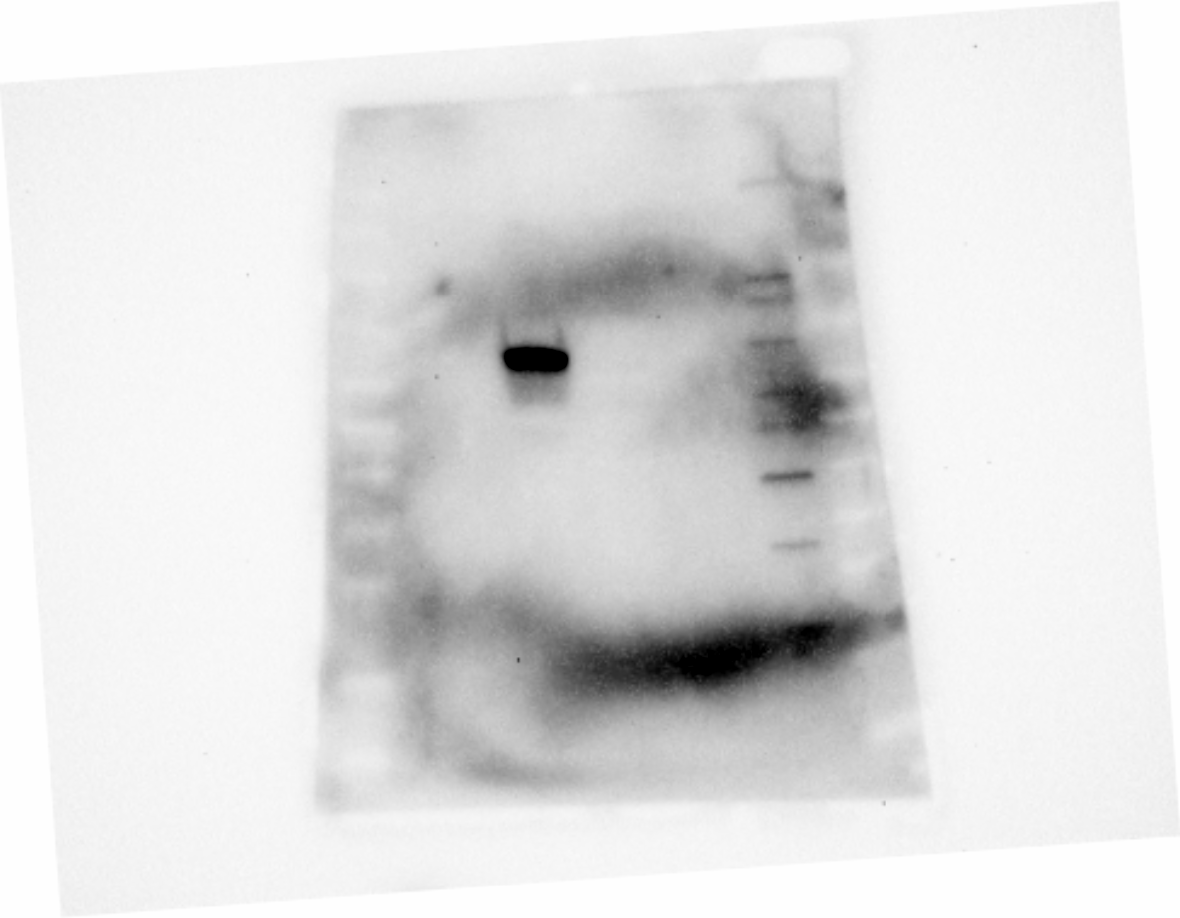

Supplement: Figure 6—source data 1. — Figures with the uncropped blots are clearly labeled with the relevant bands. [file elife-83189-fig6-data1.zip › Figure 6-source data 1/Fig. 6C eIF2D.tif]

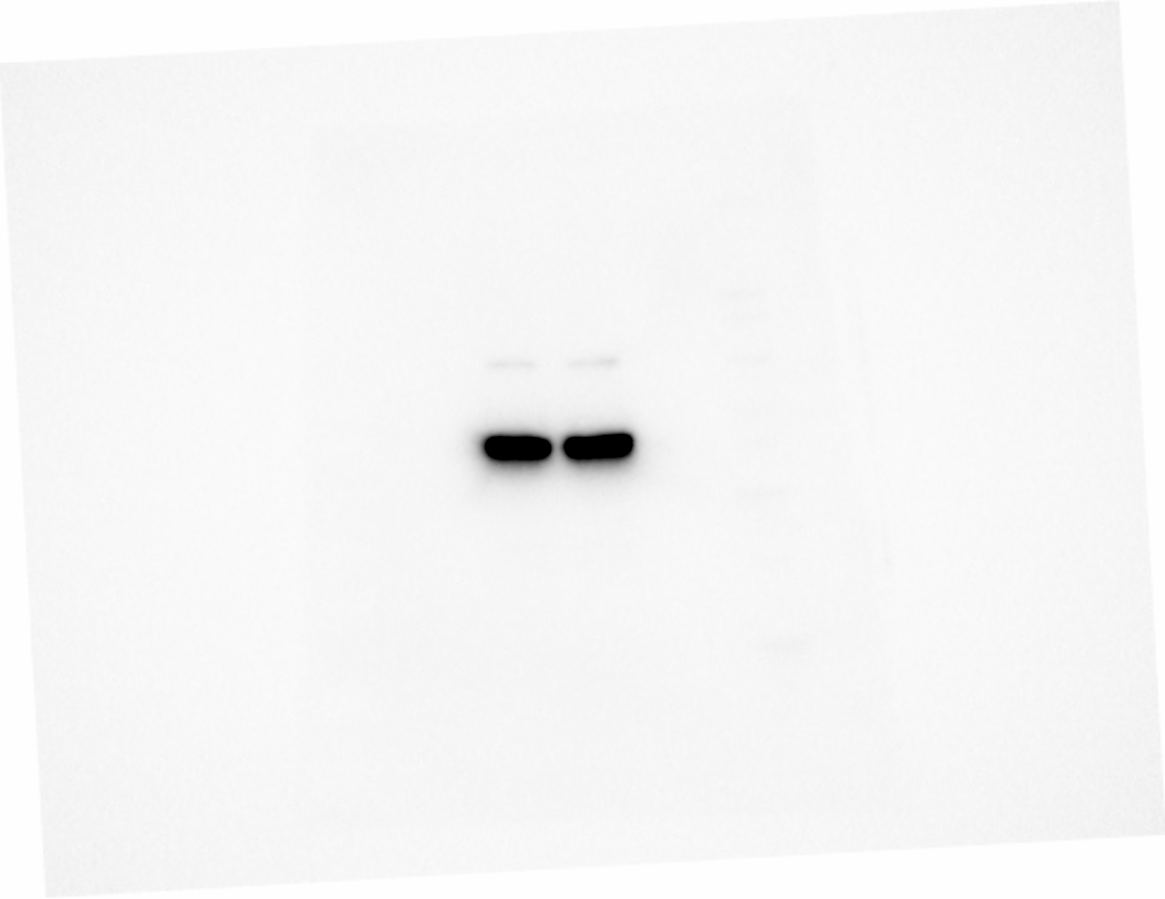

Supplement: Figure 6—source data 1. — Figures with the uncropped blots are clearly labeled with the relevant bands. [file elife-83189-fig6-data1.zip › Figure 6-source data 1/Fig. 6C a-tubulin.tif]

Figure 7 – figure supplement 1

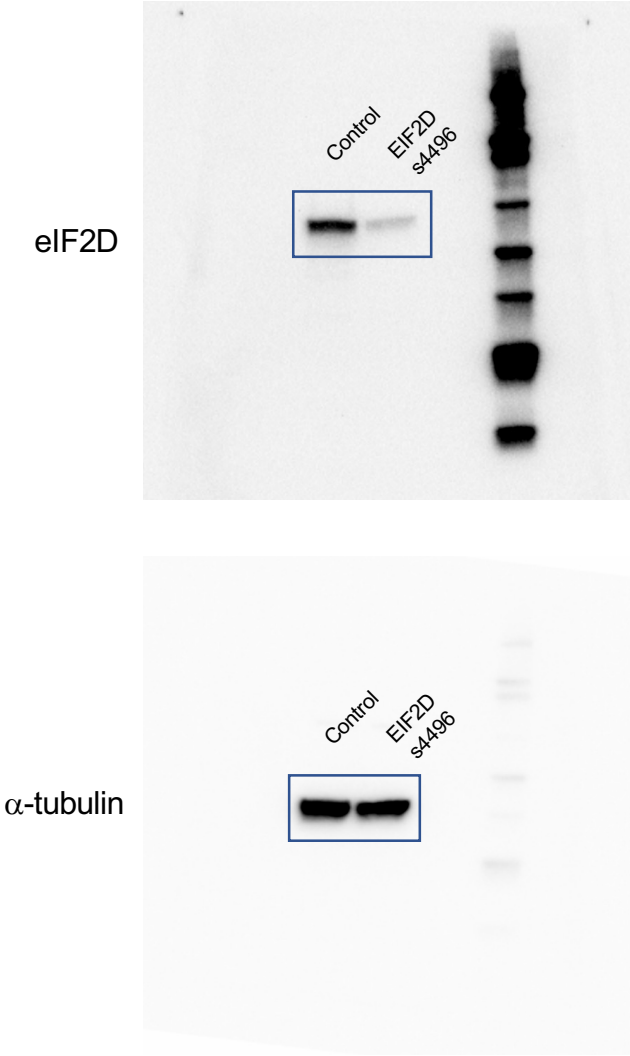

Supplement: Figure 7—figure supplement 1—source data 1. — Figures with the uncropped blots are clearly labeled with the relevant bands. [file elife-83189-fig7-figsupp1-data1.zip › Figure 7-figure supplement 1-source data 1/Figure 7 - figure supplement 1 source data labelled.pdf]

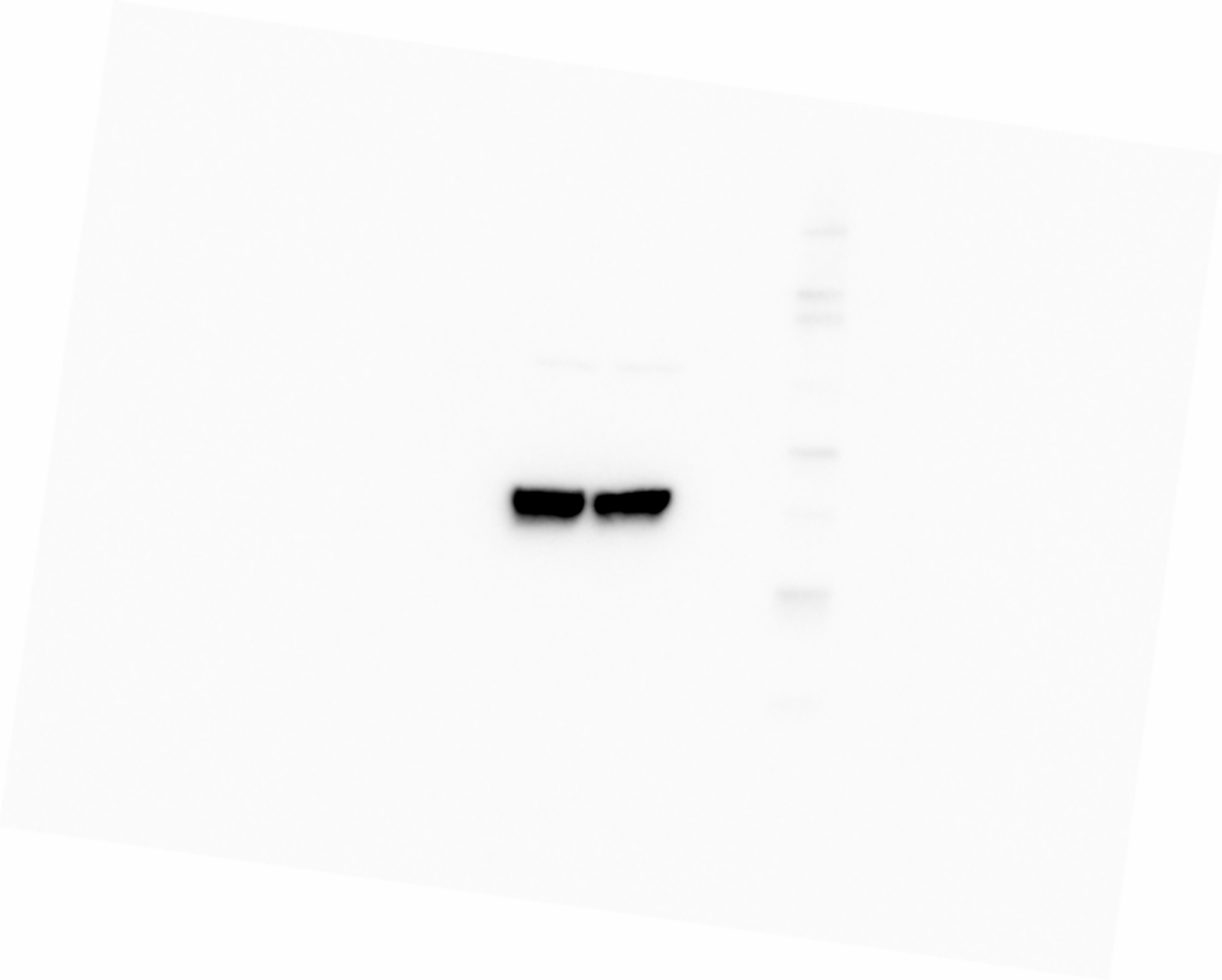

Supplement: Figure 7—figure supplement 1—source data 1. — Figures with the uncropped blots are clearly labeled with the relevant bands. [file elife-83189-fig7-figsupp1-data1.zip › Figure 7-figure supplement 1-source data 1/Fig. 7 Suppl 1 a-tubulin.tiff]

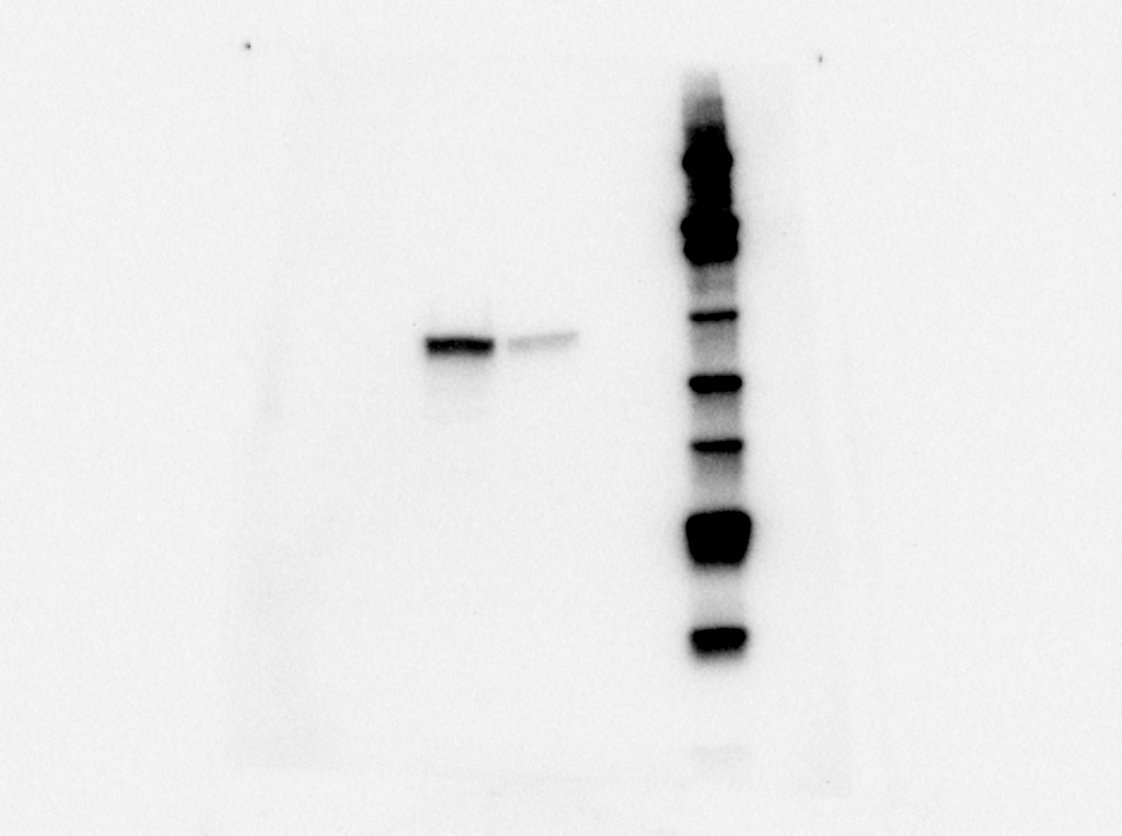

Supplement: Figure 7—figure supplement 1—source data 1. — Figures with the uncropped blots are clearly labeled with the relevant bands. [file elife-83189-fig7-figsupp1-data1.zip › Figure 7-figure supplement 1-source data 1/Fig. 7 Suppl 1 eIF2D.tiff]
