# Supplementary material for: Translation of dipeptide repeat proteins in C9ORF72 ALS/FTD through unique and redundant AUG initiation codons
Source: eLife. 2023 Sep 7;12:e83189. doi: 10.7554/eLife.83189 (PMC10541178; doi:10.7554/eLife.83189)

Figure 1 – figure supplement 2B

Poly-PR

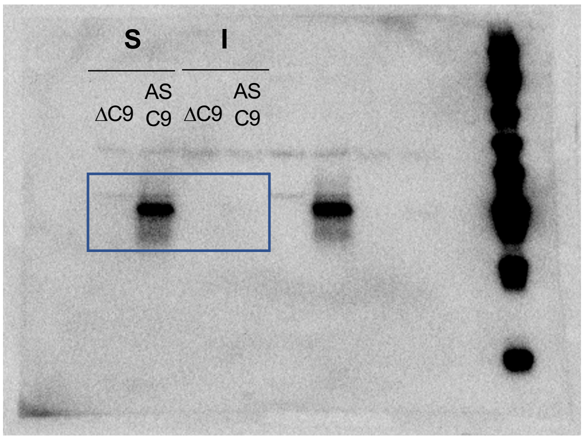

α-tubulin

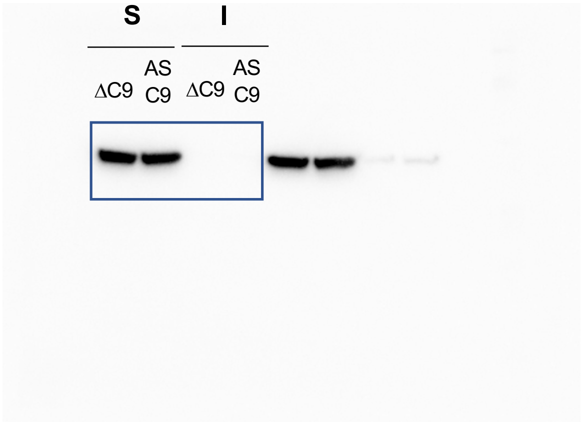

H3K4me2

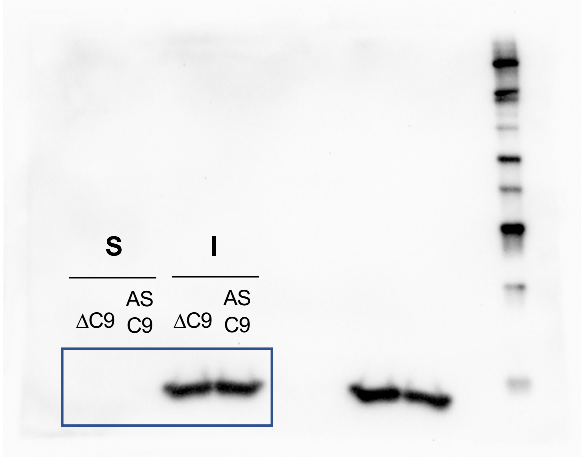

Figure 1 – figure supplement 2C

Poly-PR

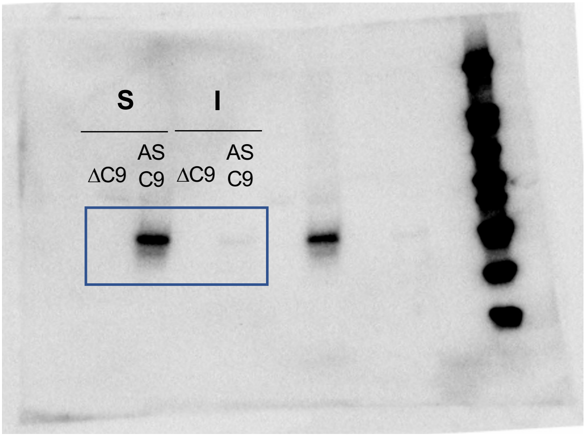

α-tubulin

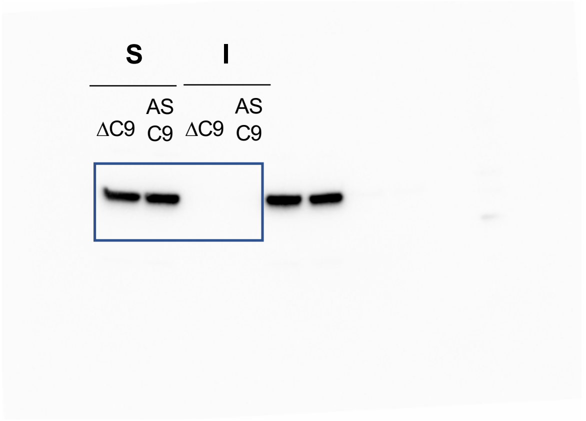

H3K4me2

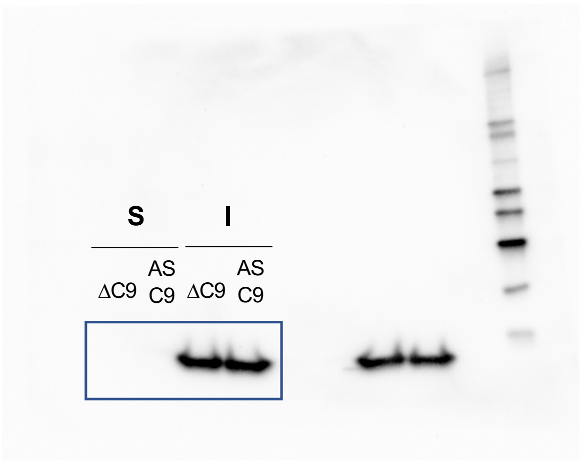

Figure 1 – figure supplement 2F

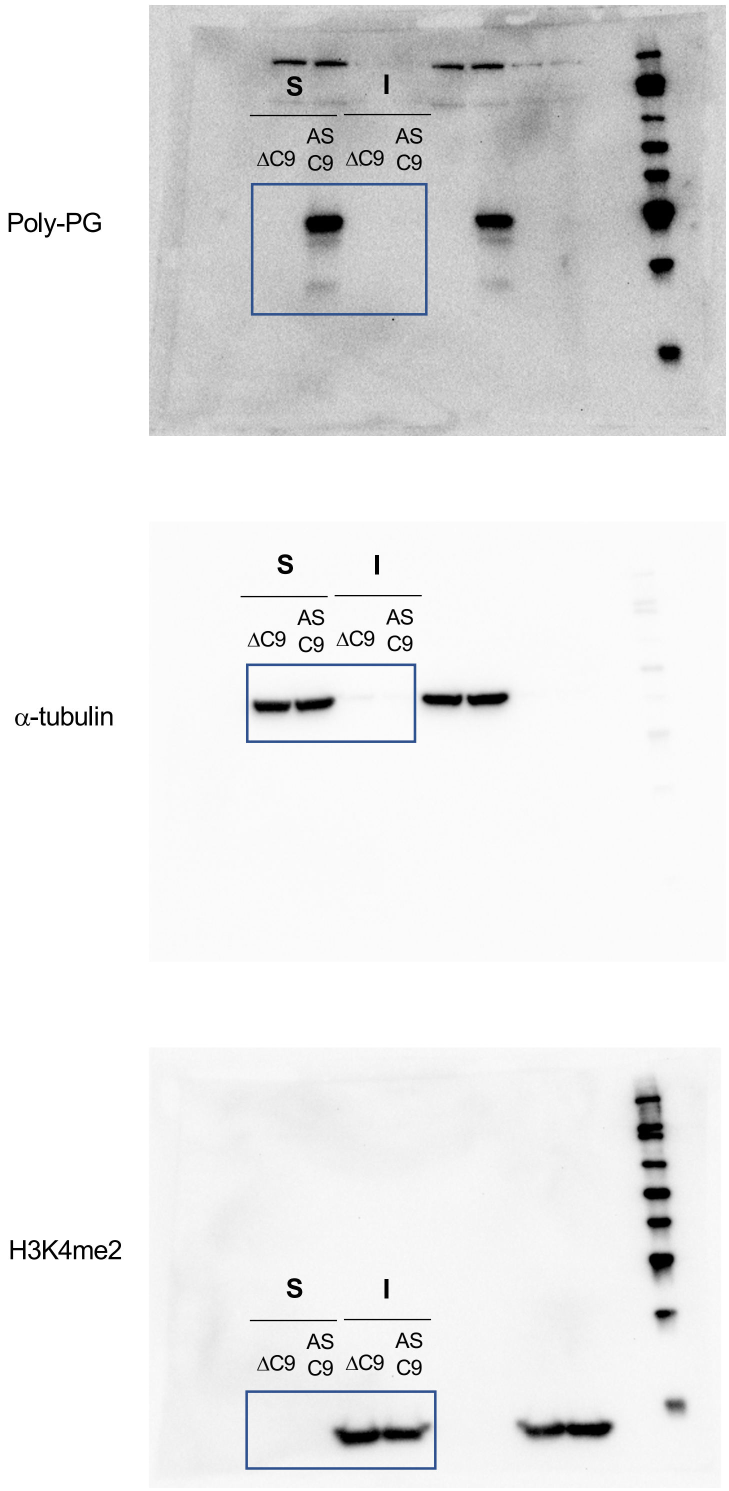

Figure 1 – figure supplement 2G

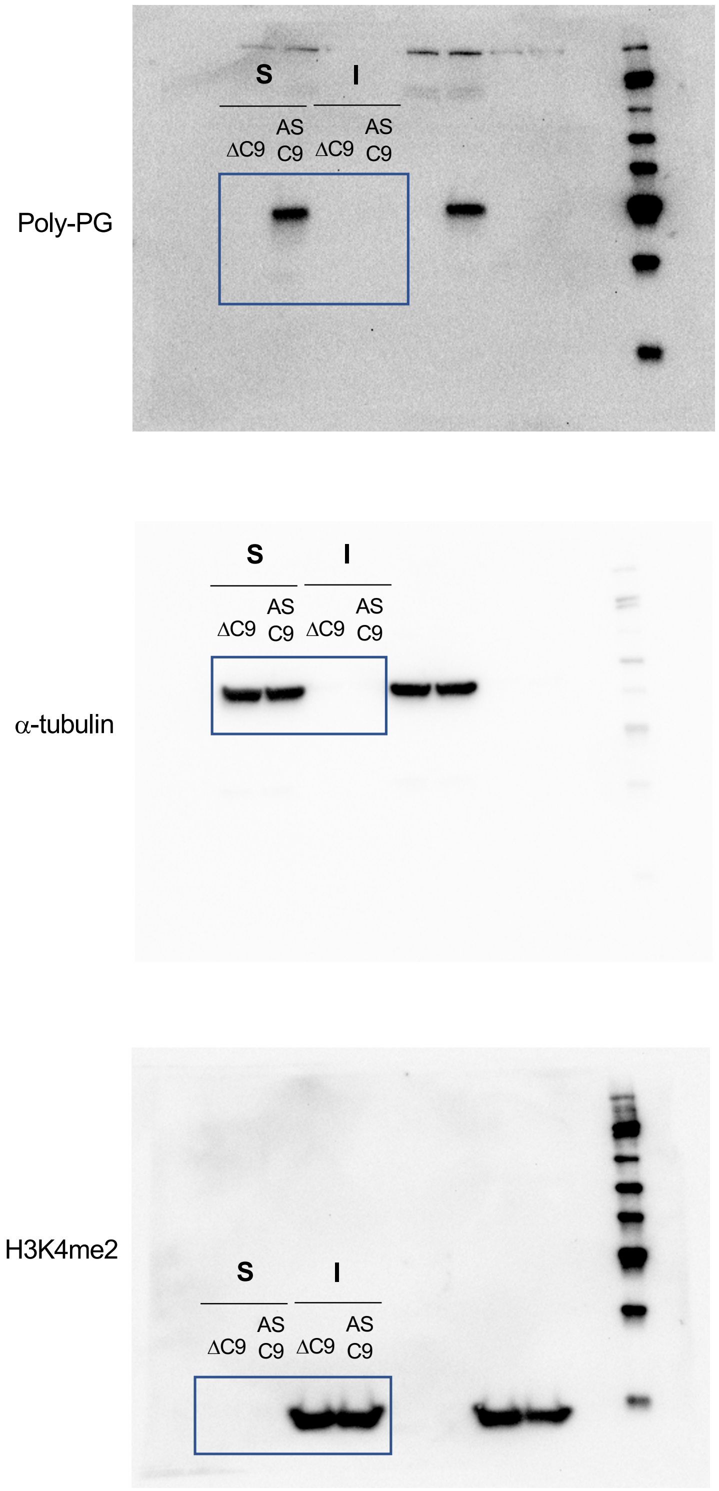

Supplement: Figure 1—figure supplement 2—source data 1. — Figures with the uncropped blots are clearly labeled with the relevant bands. [file elife-83189-fig1-figsupp2-data1.zip › Figure 1-figure supplement 2-source data 1/Figure 1 - figure supplement 2-source data labelled.pdf]
